# Social cues drive the evolution of plastic pathogen virulence in spatially and temporally variable environments

**DOI:** 10.1073/pnas.2526952123

**Published:** 2026-06-02

**Authors:** Ryuichi Kumata, Sébastien Lion, Edze R. Westra, Sylvain Gandon

**Affiliations:** ^a^CEFE, CNRS, Univ Montpellier, EPHE, IRD, Montpellier 34293, France; ^b^ Environment and Sustainability Institute, Centre for Ecology and Conservation, University of Exeter, Penryn Campus, Penryn TR10 9FE, United Kingdom

**Keywords:** plasticity, social cues, virulence, spatial structure, temporal fluctuation

## Abstract

Host–pathogen interactions are often characterized by spatial and temporal fluctuations in host density. Such variability may promote the evolution of phenotypic plasticity in pathogen virulence. However, it remains unclear how pathogens can reliably detect changes in host density. Here we show that some pathogens may exploit social cues, such as the presence of infected hosts, to gain indirect information about transmission opportunities when the density of susceptible hosts fluctuates. Our analysis demonstrates that virulence plasticity does not evolve in constant environments, but temporal and spatial variability can promote the evolution of reduced virulence in pathogens capable of detecting infected hosts. Selection for plasticity arises from the covariance between social cues and the availability of susceptible hosts. We compare the effects of temporal variation and spatial structure on the evolution of plasticity and clarify the ecological conditions under which communication systems based on social cues can emerge among viruses and other microbes. More broadly, this work provides a theoretical framework for understanding the adaptive value of plastic life-history strategies in pathogens facing variable environments.

## Significance

Pathogens experience environments that change across space and time, and successful transmission depends on responding to these changes. We show that pathogens can use social cues–the presence of other infected hosts–as indirect information about the availability of susceptible hosts. Our analysis identifies the ecological conditions under which such cue-dependent plastic strategies evolve. By linking host population dynamics to the evolution of phenotypic plasticity, this work provides a theoretical framework for understanding how environmental variability drives the evolution of complex pathogen life-history strategies.

The evolution of pathogen life-history traits has important implications for epidemiology, medicine, and agriculture (1, 2). In particular, pathogen virulence (i.e. disease-induced host mortality) is often discussed within the general theoretical framework of life-history evolution (3–5). Classical models of pathogen evolution have shown that, in spite of a direct selection against virulence, this trait can be selected for if it covaries with other positively selected life-history traits like higher transmission and lower recovery rates (6–8). Models of virulence evolution have been extended to account for the effect of different sources of heterogeneity induced by variation in quality among hosts (9–12), by spatial structure (13–17) or by temporal fluctuations of the environment (18, 19).

Classical models of pathogen evolution, however, often rely on the assumption that the life-history strategies are fixed and do not change with the environment. Yet, an increasing number of studies show that some pathogens are able to adopt plastic life-history strategies where the phenotype of the pathogen varies with some component of the environment (20, 21). This phenotypic plasticity can be adaptive for the pathogen if the modulation of its phenotype allows it to track a change of the optimal phenotype in a different environment. For instance, if the pathogen can keep track of the time since the start of the infection, it can use this as a cue to vary investment in transmission over the course of the infection (22, 23). Another form of pathogen plasticity involves the ability to detect a change in transmission opportunities. For instance, avian malaria parasites have been found to invest more in transmission when mosquitoes are around (21) or when they are more active during the day (24). This implies that avian malaria has evolved unknown biological mechanisms to perceive the presence of biting mosquitoes or to track the time of the day. More generally, all pathogens are selected to invest into transmission when more susceptible hosts are around but most pathogens do not have access to reliable cues within the host carrying information about between-host opportunities of transmission. In the present paper, we show how some pathogens can use social cues–the presence of other infected hosts–to adopt plastic virulence and transmission strategies.

The evolution of plasticity is known to be driven by the correlation between the environmental cue used to modulate the phenotypic trait and the selection imposed by a variation of the environment (25–29). A cue can be “social” when its production by one individual alters the phenotype of another individual receiving it (30). For instance, the perception of the infection status of its neighbors may affect the phenotype of a focal individual. For example, some temperate phages are carrying the Arbitrium system and sense small peptides released by prior infections and switch adaptively between lytic (virulent and highly transmissible) and lysogenic states (avirulent and less transmissible) (31–33). Importantly, this socially driven type of phenotypic plasticity is not unique to phages but is widely observed across a broad range of microbes and pathogens that communicate among conspecifics to share some information about their environment (34–37). Yet, we currently lack a theoretical framework to study the evolution of such socially driven phenotypic plasticity. Since social cues result from population dynamics, they are expected to carry imperfect information on the environment. A few theoretical models tailored to the life history of Arbitrium have revealed that temporal variation in transmission chance promotes the evolution of plasticity (33, 38, 39). However, the complexity of the ecological dynamics generated by the transient accumulation of a signal in the environment prevents a more general analysis of the ecological conditions promoting this plasticity.

Here, we explore the evolution of the plasticity of key life-history traits of pathogens—virulence and transmission—when the pathogens are able to perceive the infection of other hosts and to use this information as a social cue. We use a classical epidemiological model to study both the dynamics of the infection and the dynamics of the social cue. Building on evolutionary theory in class-structured populations (18, 40), we model the evolutionary epidemiology of pathogens that conditionally adjust their virulence when they perceive the presence of infected hosts. We derive evolutionarily stable levels of plastic and nonplastic virulence strategies in three scenarios i) a constant and well-mixed population, ii) a temporally fluctuating population, and iii) a spatially structured population. We identify the conditions promoting the evolution of virulence plasticity, and we highlight the importance of both temporal or spatial fluctuations of the environment. These fluctuations generate a covariance between the perception of infected hosts and the availability of susceptible hosts, key information that can drive the evolution of plasticity. We also highlight the selective pressures emerging in spatially structured environments that are absent in a well-mixed population. Our findings provide a theoretical framework for understanding how the interplay between environmental variation and epidemiological dynamics influences the evolution of socially driven pathogen plasticity.

## Model and Result

### Evolution in a Constant and Well-Mixed Population.

**Fig. 1.**
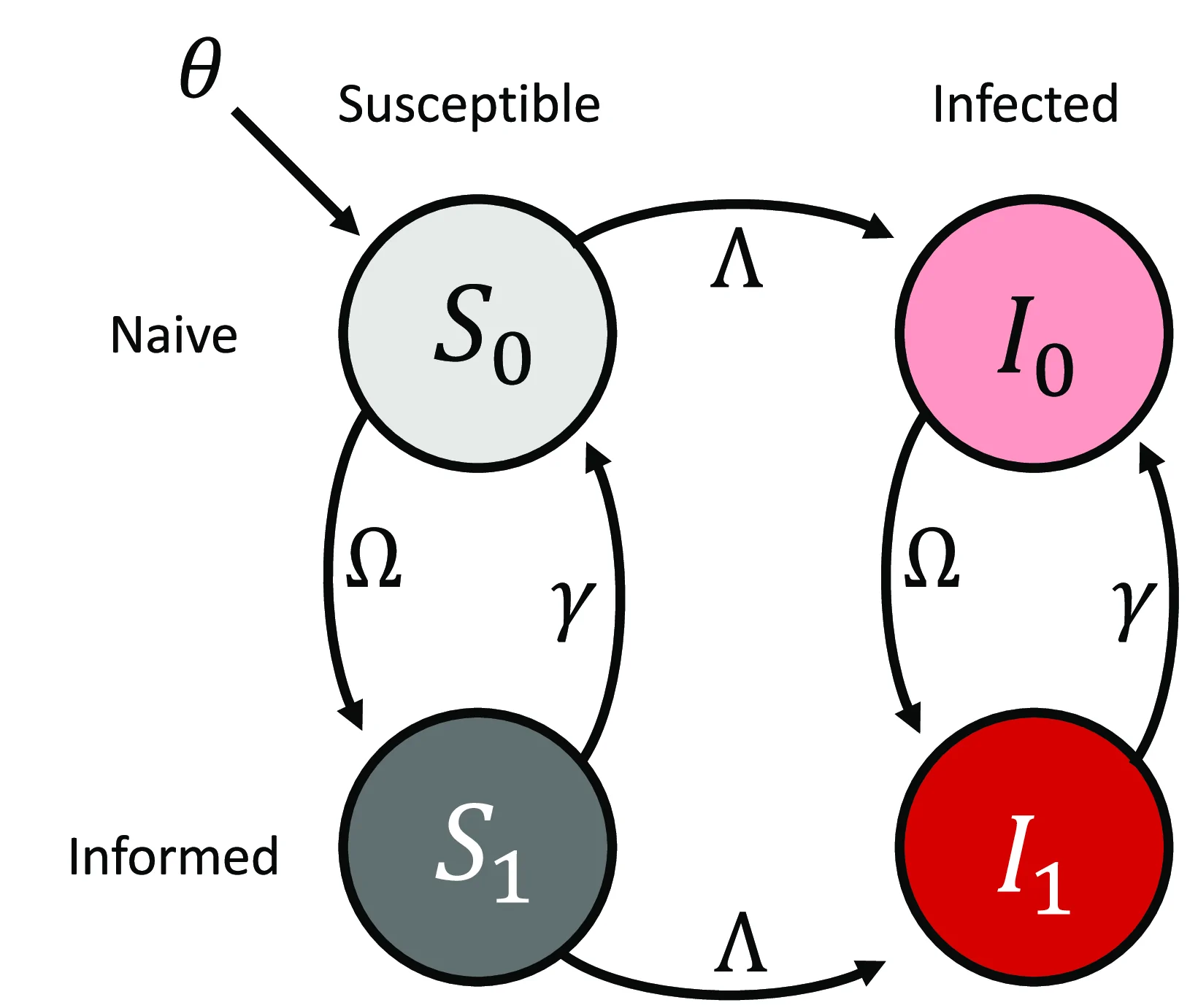
Graphical diagram for the dynamics of the infection and for the dynamics of information. The spread of the infection is driven by the force of infection $\Lambda =\beta_{0}I_{0}+\beta_{1}I_{1}$ and the death rate of infected hosts ($d+\alpha_{i}$). The spread of information is driven by the force of information $\Omega =\omega (I_{0}+I_{1})$ and the loss of information γ. For clarity, we do not represent reproduction and death rates of the hosts in this diagram.

We develop a simple epidemiological model incorporating the transmission of information among infected hosts (Fig. 1). Infected hosts produce social cues that are transmitted to other hosts. When a “naive” host perceives a social cue it becomes “informed,” but this informed state is assumed to be transient and the “informed” host can forget the information and return to the “naive” state. We denote the densities of susceptible and infected hosts as $S_{i}$ and $I_{i}$, respectively. Here, i=0 refers to naive individuals and i=1 to informed individuals. Naive susceptible hosts ($S_{0}$) are supplied to a focal population through a constant influx from the outside at rate θ, and all susceptible hosts ($S=S_{0}+S_{1}$) give birth at a per-capita rate r(1−N) to naive uninfected hosts, where N=S+I, to account for density-dependent reproduction. All uninfected hosts die at a natural rate d. Infected hosts in class i transmit pathogens to susceptible hosts at a per-capita rate $\beta_{i}$, and die at rate $d+\alpha_{i}$, where $\alpha_{i}$ is pathogen virulence (mortality induced by the infection). Information is transmitted from infected hosts to naive hosts at a per capita rate ω, and lost at rate γ. The above life cycle leads to the following dynamics (Fig. 1):

[1] $$\begin{array}{c} \begin{array}{cc} \frac{dS_{0}}{\mathrm{dt}} & =\theta +rS\left(1-N\right)-\Lambda S_{0}-\Omega S_{0}-dS_{0}+\gamma S_{1}\\ \frac{dS_{1}}{\mathrm{dt}} & =-\Lambda S_{1}+\Omega S_{0}-dS_{1}-\gamma S_{1}\\ \frac{dI_{0}}{\mathrm{dt}} & =\Lambda S_{0}-\Omega I_{0}-\left(d+\alpha_{0}\right)I_{0}+\gamma I_{1}\\ \frac{dI_{1}}{\mathrm{dt}} & =\Lambda S_{1}+\Omega I_{0}-\left(d+\alpha_{1}\right)I_{1}-\gamma I_{1}, \end{array} \end{array}$$

where $\Lambda =\beta_{0}I_{0}+\beta_{1}I_{1}$ is the rate of infection of susceptible hosts (the force of infection) and $\Omega =\omega (I_{0}+I_{1})$ is the rate of transmission of information to naive hosts (the force of information). The basic reproduction ratio of the pathogen is $R_{0}=\beta_{0}S_{\emptyset }/\left(d+\alpha_{0}\right)$, where $S_{\emptyset }$ is the equilibrium density of susceptible hosts in the absence of infection. When $R_{0}>1$ the pathogen can invade the uninfected host population and, ultimately, the system reaches an equilibrium point $\hat{E}=\left\{{\hat{S}}_{0},{\hat{S}}_{1},{\hat{I}}_{0},{\hat{I}}_{1}\right\}$ (*SI Appendix*, section 2).

Next, we analyze the evolution of the pathogen under the assumption that virulence has an effect on pathogen transmission: Parasites with higher virulence tend to transmit more efficiently but have shorter infection duration (41). Specifically, we assume that the trade-off function β(α) is increasing ($\beta^{\prime }\left(\alpha \right)>0$) and saturating ($\beta^{\prime \prime }\left(\alpha \right)<0$)—a classical assumption of previous virulence evolution theory (41). We first investigate the evolution of nonplastic virulence strategies, where naive and informed hosts have equal virulences ($\alpha_{0}=\alpha_{1}=\alpha$), in contrast to plastic virulence strategies where virulence differs between these two classes of hosts ($\alpha_{0}\neq \alpha_{1}$). We analyze the fate of a rare mutant strain introduced in a host population infected by a wild-type pathogen at its endemic equilibrium $\hat{E}$ (42, 43). Under the assumption that the phenotypic effect of the mutation is small ($\alpha^{m}=\alpha +\epsilon$ and ϵ≪1), we show in *SI Appendix*, section S2A that the direction of selection on nonplastic virulence is given by the selection gradient

[2] $$\begin{array}{c} S=\beta^{\prime }\left(\alpha \right)\hat{S}-1, \end{array}$$

where $\hat{S}={\hat{S}}_{0}+{\hat{S}}_{1}$ is the total density of susceptible hosts evaluated at the endemic equilibrium of the resident population and $\beta^{\prime }\left(\alpha \right)$ is the derivative of the trade-off function β(α). We thus recover a classical result where the fate of the mutant is determined by the beneficial effect on transmission (the first term) and the mortality cost (the second term) (41, 43). This selection gradient allows us to identify the nonplastic evolutionary stable (ES) virulence, $\alpha^{*}$, where these two forces balance each other (S=0). In our numerical analyses, we use the specific tradeoff function between transmission and virulence, $\beta \left(\alpha \right)=\beta_{\text{max}}\sqrt{\alpha }$. This tradeoff function has often been used in earlier models of virulence evolution, and it yields the nonplastic ES virulence $\alpha^{*}=d$ (*SI Appendix*, section S2A).

Next, we assume that the pathogen is able to adopt plastic virulence strategies. The direction of selection on the trait in class i is then given by the class-specific selection gradient

[3] $$\begin{array}{c} S_{i}=\underset{T_{i}}{\underbrace{\hat{f_{i}}\left(\beta^{\prime }\left(\alpha_{i}\right)\hat{S}-1\right)}}+\underset{F_{i}}{\underbrace{\Delta \rho \frac{\partial f_{1}^{m}}{\partial \alpha_{i}^{m}}}}, \end{array}$$

where $\Delta \rho =\rho_{1}-\rho_{0}$ and $\rho_{i}=\beta_{i}\hat{S}-\left(d+\alpha_{i}\right)$ is the per-capita growth rate of the parasite within class i. The first term, $T_{i}$, captures the influence of the trade-off between transmission and virulence (as in Eq. **2**) weighted by the equilibrium class frequency ${\hat{f}}_{i}$ in the wild-type infected population. The second term $F_{i}$ is the indirect effect on selection through the change of class distribution due to increasing class-specific virulence. This class-specific selection can be used to study whether plastic virulence can be selected from a nonplastic ESS. Note that, at the nonplastic ESS $\alpha^{*}$ we have $T_{i}=0$ (because Eq. **2** is zero by definition of the nonplastic ESS) and $F_{i}=0$ (because $\rho_{0}=\rho_{1}$ if pathogen virulence is the same in the two classes). Hence, we obtain $S_{i}{|}_{\alpha =\alpha^{*}}=0$, which implies that, in a constant environment, plasticity cannot evolve away from the nonplastic ESS virulence. More generally, we can show that, even starting from a plastic virulence strategy, the pathogen will evolve to the unique nonplastic ESS strategy $\alpha^{*}$ (*SI Appendix*, section 2B).

### Evolution in a Temporally Fluctuating Population.

**Fig. 2.**
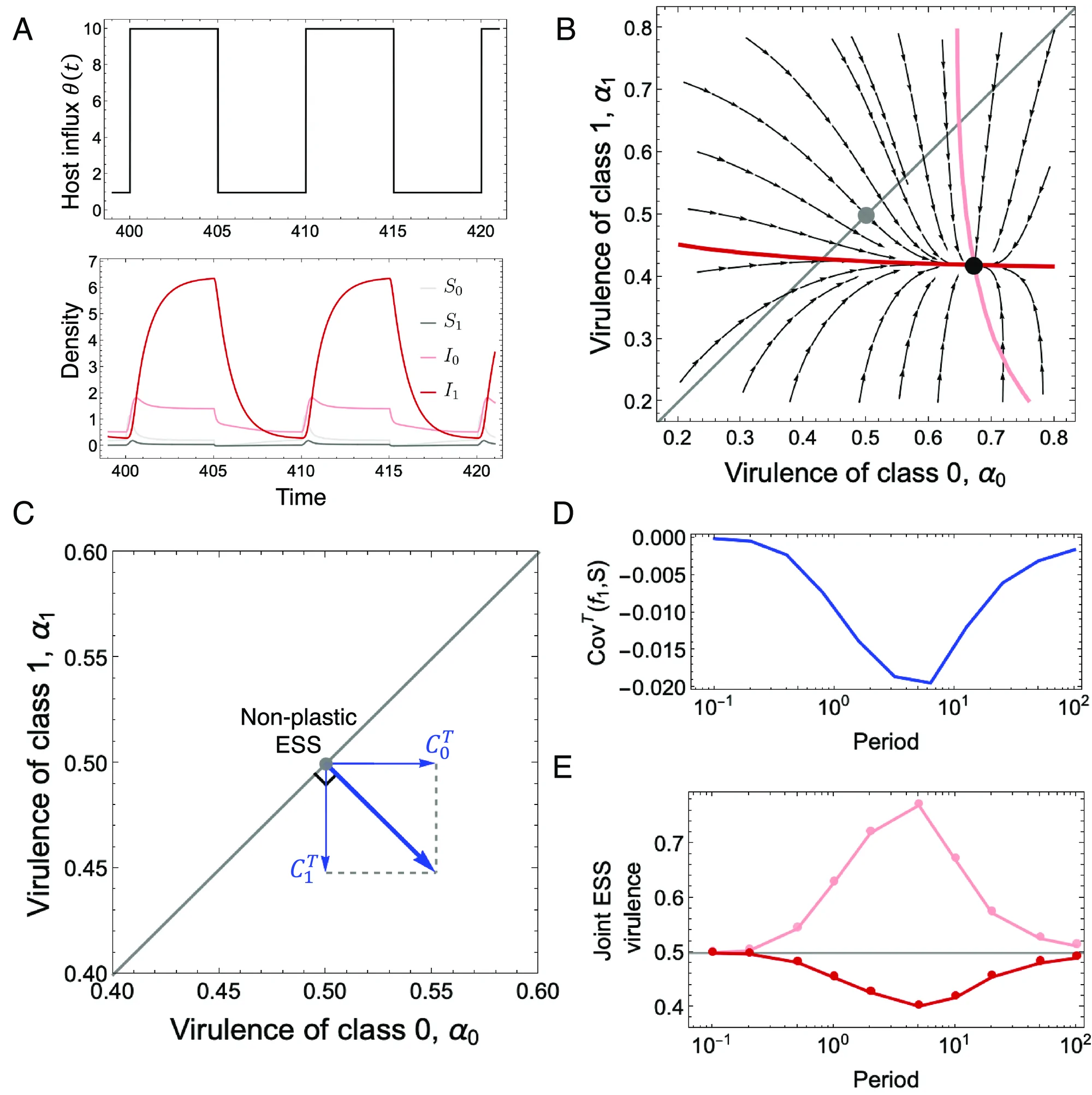
Evolution of plasticity in a temporally fluctuating population. (*A*) Epidemiological dynamics when the influx θ(t) follows a square wave varying between 1 and 10 with period T=10. (*B*) Class-specific selection gradients on virulence in naive and informed classes. The gray point is the nonplastic ESS $\alpha^{*}$ and the black point is the joint ESS $\left\{\alpha_{0}^{**},\alpha_{1}^{**}\right\}$. The arrows indicate the direction of selection, and colored lines indicate the nullcline of the selection gradient for virulence in the naive class $\alpha_{0}$ (pink) and that in the informed class $\alpha_{1}$ (red). The gray diagonal line corresponds to nonplastic virulence strategies such that $\alpha_{0}=\alpha_{1}$. (*C*) Geometric interpretation of the selection gradient acting on plasticity (blue arrows) when the pathogen adopts the nonplastic strategy (gray point). (*D*) Temporal covariance ${\text{Cov}}^{\text{T}}\left(f_{1},S\right)$ between the frequency of the informed and infected class and the density of susceptible hosts for different values of the period T. (*E*) Joint ES virulence for different values of the period T. The line is numerically calculated from the selection gradient for $\alpha_{0}$ (pink) and $\alpha_{1}$ (red) (Eq. **4**) while the point is the averaged value of the evolved trait distribution from evolutionary simulations (*SI Appendix*, section 4F). The variance of the evolved distribution, which is produced solely by mutation–selection balance, is omitted for clarity of the figure. We used the specific trade-off function $\beta \left(\alpha \right)=\beta_{\max}\sqrt{\alpha }$. Parameter values: $\beta_{\max}=5,\omega =1,\gamma =1,r=1,$ and d=0.5.

Previous studies have shown that periodic environmental fluctuations can favor the evolution of plastic strategies (21, 33, 38, 39). In this section, we assume that environmental fluctuations are driven by periodic fluctuations of the host influx θ(t), with period T. This periodic influx of susceptible hosts generates periodic epidemics and, ultimately, the system converges to a periodic attractor (Fig. 2*A* and *SI Appendix*, section 3).

We use the approach developed by refs. 18 and 44 to obtain the selection gradient for class-specific virulence on the periodic attractor

[4] $$\begin{array}{c} \begin{array}{cc} S_{i}^{\text{T}}= & \underset{T_{i}^{\text{T}}}{\underbrace{\left\langle f_{i}\right\rangle \left(\beta^{\prime }\left(\alpha_{i}\right)\left\langle S\right\rangle -1\right)}}+\underset{F_{i}^{\text{T}}}{\underbrace{\langle \Delta \rho \frac{\partial f_{1}^{m}}{\partial \alpha_{i}^{m}}\rangle }}\\ & +\underset{C_{i}^{\text{T}}}{\underbrace{\beta^{\prime }\left(\alpha_{i}\right){\text{Cov}}^{\text{T}}\left(f_{i},S\right)}}, \end{array} \end{array}$$

where ⟨X⟩ is the temporal average (arithmetic mean) of the dynamical variable X(t) over one period when the system sits on the periodic attractor. The selection gradient, $S_{i}^{\text{T}}$, can be decomposed into three components. The first term, $T_{i}^{\text{T}}$, is the product of the average class frequency and the average trade-off between transmission and virulence. The second term, $F_{i}^{\text{T}}$, is the temporal average of the indirect selective force which arises from the change in class distribution. The third term, $C_{i}^{\text{T}}$, is new (compare Eqs. **3** and **4**) and emerges from the temporal covariance between the class frequency of infected hosts $f_{i}\left(t\right)$ and the density of susceptible hosts S(t). We can use Eq. **4** to show that, in our model, $\alpha^{*}$, which is defined in the previous section, remains the nonplastic ESS in a fluctuating environment (*SI Appendix*, section 3A). Next, we use the selection gradients for class-specific virulence (Eq. **4**) to study pathogen evolution toward a joint evolutionary stable strategy (joint ESS) at the intersection of the evolutionary nullclines $S_{0}^{T}=0$ and $S_{1}^{T}=0$ (Fig. 2*B*). This analysis shows that the joint ESS ($\alpha_{0}^{**}$, $\alpha_{1}^{**}$) (black point in Fig. 2*B*) differs from the nonplastic ESS ($\alpha^{*}$) (gray point in Fig. 2*B*). Crucially, the evolution of plasticity favors lower virulence in informed hosts and higher virulence in naive hosts ($\alpha_{1}^{**}<\alpha^{*}$ and $\alpha_{0}^{**}>\alpha^{*}$).

What factors drive the evolution of plasticity in temporally fluctuating populations? To answer this question we focus on the scenario where the pathogen adopts the nonplastic ESS virulence strategy $\alpha_{0}=\alpha_{1}=\alpha^{*}$ (gray dot in Fig. 2*C*). At this point we have $T_{i}^{\text{T}}=F_{i}^{\text{T}}=0$ and the selection gradient reduces to ${\left.S_{i}^{\text{T}}\right|}_{\alpha =\alpha^{*}}=C_{i}^{\text{T}}$. Fig. 2*C* provides a geometrical decomposition of the selection gradient $C^{\text{T}}$ in trait space. Note that this selection gradient intersects orthogonally with the diagonal at the nonplastic ESS, because selection acting in the two classes of hosts (naive and informed) have the same magnitude but opposite signs [indeed, $C_{0}^{\text{T}}=-C_{1}^{\text{T}}$, because $f_{0}\left(t\right)=1-f_{1}\left(t\right)$]. In addition, extensive numerical computation of the covariance $C_{1}^{\text{T}}$ suggests it is always negative (Fig. 2*D*). Indeed, the epidemiological dynamics shown in Fig. 2*A* indicates that S(t) reaches a maximal value soon after the start of a large host influx and declines toward an equilibrium level. $I_{0}\left(t\right)$ reaches a maximal value soon after S(t) but $I_{1}\left(t\right)$ peaks much later. Consequently, the density of susceptible cells is expected to be relatively low when many infected hosts are in the informed state. In other words, the negative covariance between the perception of the informed state and the density of susceptible hosts implies that the informed state can be used as a social cue to infer the scarcity of susceptible hosts. When susceptible hosts are abundant, transmission opportunities are high, which favors high-transmission strategies and higher virulence. Conversely, when susceptible hosts are scarce, transmission opportunities are limited, and selection favors reduced transmission and lower virulence. Because the informed state is negatively correlated with S(t), it can be used as a signal to promote the evolution of a lower virulence strategy.

Note that the above results are robust when we use another periodic function θ(t) to model the fluctuations of the influx of susceptible hosts (*SI Appendix*, section 3B). Interestingly, when θ(t) fluctuates as a sine-wave with period T and amplitude δ, we obtained an approximation of the covariance $C_{1}^{\text{T}}$ which allows us to explore the effects of various parameters of the model on the magnitude of the covariance. In particular, we find that the magnitude of the covariance is maximized for intermediate values of the period T (Fig. 2*D* and *SI Appendix*, Fig. S4). Indeed, when environmental fluctuations are fast (T→0), the population dynamics of the pathogen cannot track the environment, and the covariance vanishes. Conversely, when environmental fluctuations are slow (T→∞), the epidemiological dynamics has time to reach its endemic equilibrium before a change in the environment. However, the equilibrium density of susceptible hosts $\hat{S}$ is independent of θ, so that there is no correlation between information and the density of susceptible hosts, and the covariance vanishes. Interestingly, the magnitude of the covariance also depends on parameters governing the speed of the epidemiological dynamics (β and α) and the speed of the information dynamics (ω and γ). The magnitude of the covariance is maximized when the speeds of these two dynamics are similar (*SI Appendix*, section 3B and Fig. S4) and when the natural death rate d is intermediate (*SI Appendix*, section 3B and Fig. S5).

In summary, we find that plasticity in virulence can evolve in a fluctuating environment and that it is driven by the sign and the magnitude of the covariance $C_{1}^{\text{T}}$. First, because this covariance is negative, virulence evolves lower values in informed hosts than in naive ones: $\alpha_{1}^{**}<\alpha^{*}<\alpha_{0}^{**}$. Second, the difference $\alpha_{1}^{**}-\alpha_{0}^{**}$ (the amplitude of plasticity at the joint ESS) increases with the covariance which is maximized for intermediate speed of environmental fluctuations relative to the speed of the epidemic and of information spread (Fig. 2*E*).

### Evolution in a Spatially Structured Population.

**Fig. 3.**
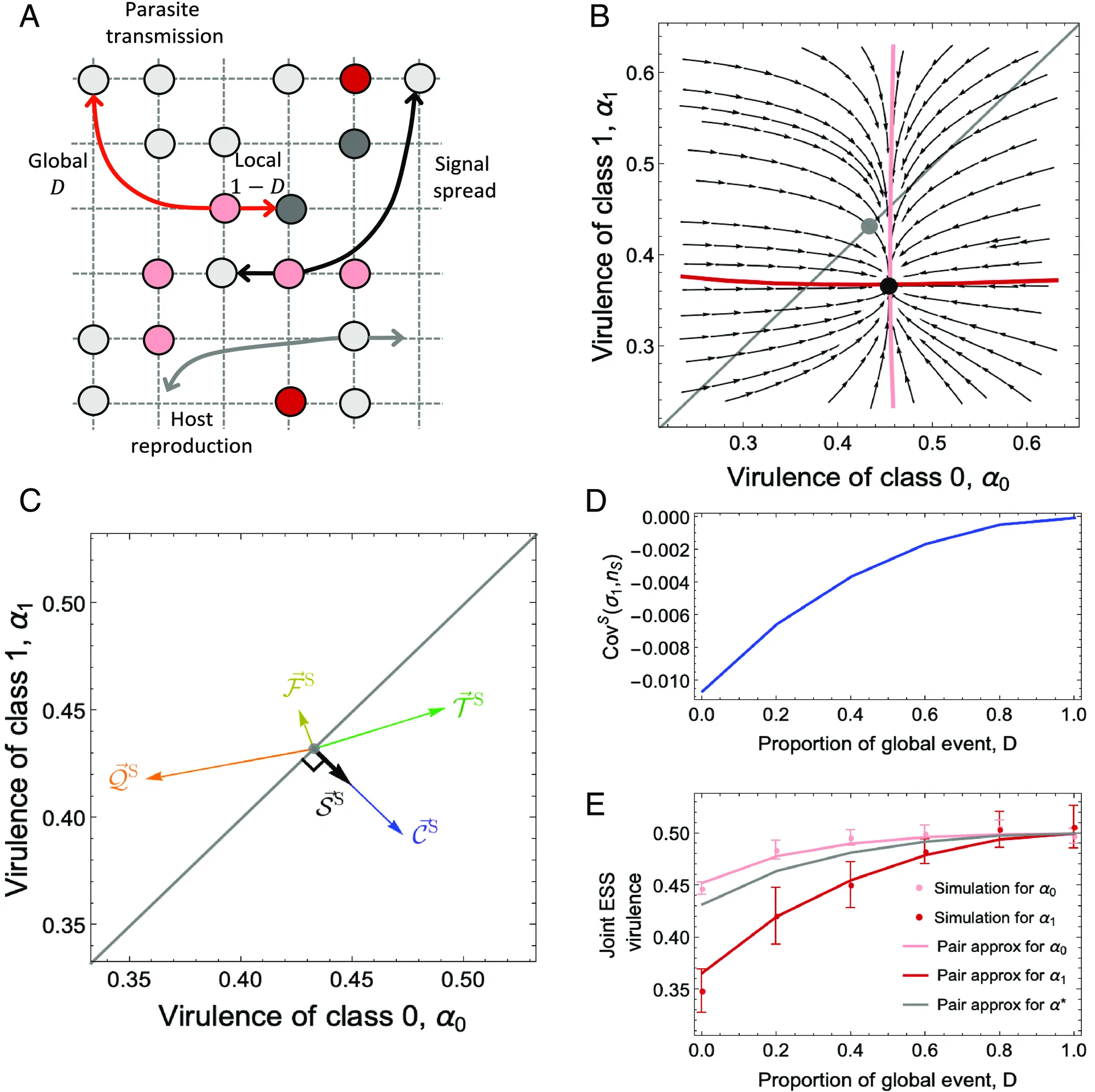
Evolution of plasticity in a spatially structured population. (*A*) Graphical illustration of the dynamics on the lattice (we use the same color legend as in Fig. 1). (*B*) We plot the selection gradient for class-specific virulence obtained by pair approximation. As in Fig. 2, pink and red lines show the evolutionary nullclines for $\alpha_{0}$ and $\alpha_{1}$ respectively. The gray line correspond to nonplastic strategies such that $\alpha_{0}=\alpha_{1}$ and the gray point is the nonplastic ES virulence $\alpha^{*}$. The black point indicates the joint ESS virulence $\left\{\alpha_{0}^{**},\alpha_{1}^{**}\right\}$. (*C*) Geometric decomposition of the different components of the selection gradient (Eq. **5**) at the nonplastic ESS. (*D*) Spatial covariance between information and local susceptible density at the nonplastic ESS $\alpha^{*}$, as calculated by the pair approximation (*SI Appendix*, section 6). (*E*) Average virulence (± SD) obtained from 10 independent simulations for different values of dispersal D (*SI Appendix*, section 4F). Lines indicate the values of nonplastic ESS $\alpha^{*}$ (gray) and joint ESS of $\alpha_{0}^{**}$ (pink) and $\alpha_{1}^{**}$ (red) expected from Eq. **5** and pair approximation. We used the specific trade-off function $\beta \left(\alpha \right)=\beta_{\max}\sqrt{\alpha }$. Parameter values: $r=10,\beta_{\max}=5,d=0.5,\omega =1$, and γ=1.

In this section, we examine how spatial structure influences the evolution of virulence plasticity. To incorporate spatial structure we assume that the dynamics of the system takes place on a square lattice where each site is either empty or occupied by a single host ($S_{0}$, $S_{1}$, $I_{0}$, or $I_{1}$). Pathogen transmission, the spread of information, and host reproduction are all influenced by spatial structure (Fig. 3*A*). To modulate the degree of spatial structure, we assume that these events can occur either globally, with probability D, or locally, with probability 1−D (13). For instance, global transmission to a susceptible host depends on the global density of susceptible hosts $p_{S}$ (i.e. the fraction of sites occupied by a susceptible host on the lattice), while local transmission depends on the fraction of susceptible hosts in the neighborhood of a focal site. Averaged over all focal sites, this gives the local density $q_{S/I_{i}}$ of susceptible hosts in the neighborhood of an infected host in class i. When D=1, all events are global, and we recover the dynamics of the well-mixed system (Eq. **1**) with θ=0. At the other extreme, when D=0, all events are fully local and the spatial structure is maximal. In the following, we assume no extrinsic influx of susceptible hosts (θ=0) because we want to focus here on the effect of spatial structure on plasticity evolution without an external source of temporal variation. In *SI Appendix*, section 4G, however, we explore more complex scenarios with a periodic influx of susceptible hosts in a spatially structured population.

We use spatial moment equations to model the spatial dynamics of our class-structured population (45–47). We show in *SI Appendix*, section 4C that the direction of selection on virulence in class i is given by the following class-specific selection gradient

[5] $$\begin{array}{c} \begin{array}{cc} S_{i}^{\text{S}} & =\underset{T_{i}^{\text{S}}}{\underbrace{f_{i}\left(\beta^{\prime }\left(\alpha_{i}\right)\left[S|I\right]-1\right)}}+\underset{F_{i}^{\text{S}}}{\underbrace{\Delta \rho \frac{\partial f_{1}^{m}}{\partial \alpha_{i}^{m}}}}\\ & +\underset{C_{i}^{\text{S}}}{\underbrace{\left(1-D\right)\beta^{\prime }\left(\alpha_{i}\right){\text{Cov}}^{\text{S}}\left(\sigma_{i},n_{S}\right)}}\\ & +\underset{Q_{i}^{\text{S}}}{\underbrace{\left(1-D\right)\sum_{j=0,1}\beta_{j}f_{j}\frac{\partial q_{S/I_{j}^{m}}}{\partial \alpha_{i}^{m}}}}, \end{array} \end{array}$$

where $\left[S|I\right]=\left(1-D\right)q_{S/I}+Dp_{S}$ is the “effective density” of susceptible hosts accessible to a pathogen in the resident population and $\Delta \rho =\rho_{1}-\rho_{0}$ where $\rho_{i}=\beta_{i}\left[S|I_{i}\right]-\left(d+\alpha_{i}\right)$ is the per-capita growth rate of infected hosts in class i in the resident population when there are no between-class transitions. The spatial selection gradient, $S_{i}^{\text{S}}$, can thus be decomposed into four interpretable components. The first term, $T_{i}^{\text{S}}$, reflects the effect of the transmission–virulence trade-off. The second term, $F_{i}^{\text{S}}$, captures the indirect selection resulting from changes in class frequencies. The third component, $C_{i}^{\text{S}}$, depends on ${\text{Cov}}^{\text{S}}\left(\sigma_{i},n_{S}\right)$ measuring the spatial covariance between the informed state, $\sigma_{i}$, and the density of locally accessible susceptible hosts, $n_{S}$. Finally, $Q_{i}^{\text{S}}$ quantifies the effect of the mutation on the local density of susceptible hosts experienced by a mutant pathogen and is therefore a measure of the strength of local competition for susceptible hosts. Note that the first three components $T_{i}^{\text{S}},F_{i}^{\text{S}}$ and $C_{i}^{\text{S}}$ are analogous to the terms derived for temporally fluctuating environments in Eq. **4**. This structural similarity underscores the generality of the underlying evolutionary forces acting on the class-specific virulence in both temporal and spatial settings. In contrast, $Q_{i}^{\text{S}}$ is a novel component that arises exclusively in spatially structured populations. In previous studies with no information structure, similar terms capturing the effect of local competition for hosts have been identified and shown to capture the effect of kin competition among pathogens (14, 15). Although we failed to derive an analytical expression for this term in our class-structured model, we expect, based on these earlier studies, that more virulent pathogens will tend to experience a lower local density of susceptible hosts, so that this last term should generally be negative and select for lower virulence (but see refs. 14 and 15).

To explore the effects of spatial structure, we carried out explicit stochastic simulations and allowed virulence to evolve independently in the different classes (e.g. naive and informed) of pathogens. In addition, we used pair approximation (46, 48) to numerically compute the different components of the selection gradient Eq. **5**, as detailed in *SI Appendix*, section 6. The evolutionary simulations based on pair approximation coincide with the explicit stochastic simulations and predict very well the evolution of plasticity in virulence (Fig. 3*B*). At the plastic ESS, we find that, as in the temporally fluctuating environment (Fig. 2*E*), virulence evolves lower values in the informed class $\alpha_{1}^{**}<\alpha^{*}<\alpha_{0}^{**}$ (Fig. 3*E*).

What factors drive the evolution of plasticity in spatially structured populations? Our geometric decomposition of the selection gradient reveals that, as in the temporally fluctuating scenario, the covariance between the informed state and the availability of susceptible hosts remains the key driver of plasticity. To illustrate this, we plot the total selection gradient and its components as two-dimensional vectors in trait space when the pathogen sits at the nonplastic ESS $\alpha^{*}$ (Fig. 3*C*). The thick black arrow represents the direction of the total selection gradient, denoted by ${\vec{S}}^{\text{S}}$, while colored arrows represent the four components of Eq. **5** denoted by ${\vec{T}}^{\text{S}},{\vec{F}}^{\text{S}},{\vec{Q}}^{\text{S}},{\vec{C}}^{\text{S}}$. We show that the covariance-related vector $\vec{C^{\text{S}}}$ points in the same direction as the total selection gradient ${\vec{S}}^{\text{S}}$, which indicates that spatial covariance between information and susceptible host density is the key driver of the evolution of plasticity, just as temporal covariance drives plasticity in temporally variable populations. To quantify this effect, we computed the spatial covariance term ${\text{Cov}}^{\text{S}}\left(\sigma_{1},n_{S}\right)$ at the nonplastic ESS across varying degrees of spatial structure (Fig. 3*D*). Numerical computation of this spatial covariance shows that it is always negative when there is some spatial structure and becomes more negative as spatial structure increases (i.e., as D decreases). This negative correlation reflects the spatial segregation of informed infected hosts and susceptible hosts, which generates directional selection pressure favoring plastic responses. Finally, we stress that the nonplastic ESS for virulence $\alpha^{*}$ (gray line) is always lower when spatial structure increases (D decreases). This lower virulence is due to the last term in Eq. **5**, which, as noted above, captures the classical effect of kin competition among pathogens arising from limited dispersal in spatially structured populations (13–15).

## Discussion

Phenotypic plasticity is expected to be a suitable adaptive response allowing organisms to cope with the variability of the environment if they can use reliable cues to detect environmental changes (25, 49, 50). Here we explore the conditions for the evolution of pathogen plasticity when the availability of susceptible hosts varies in time or space. We show that this variation changes the cost–benefit ratio associated with increased virulence (Eq. **2**) and selects for plastic life-history strategies. Importantly, the pathogen does not have a direct access to the information on the density of susceptible hosts. Yet, our analysis shows that it may be adaptive to use a social cue (here an informed state transmitted by infected hosts) if it is providing indirect information on the density of susceptible hosts. In addition, we show that the quality of this information is quantified by the covariance between the perception of this social cue and the density of susceptible hosts.

In well-mixed and temporally constant environments, this covariance is zero. As a result, the cue is not ecologically informative, and plasticity is not favored. We then recover a classical prediction of virulence evolution theory that pathogens should evolve an intermediate level of virulence which corresponds to minimizing the equilibrium density of susceptible hosts (5, 41). In contrast, temporally fluctuating or spatially structured environments promote the build up of this “meaningful” covariance, allowing the perception of a social cue to modulate the infection strategies. A negative covariance implies that the detection of the cue predicts host scarcity and therefore favors a reduction in transmission and virulence, in agreement with the general prediction that the selective benefit of virulence is proportional to the density of susceptible hosts under a transmission–virulence trade-off (see *SI Appendix*, section 2A and refs. 8 and 51).

In temporally fluctuating environments, we found that temporal covariance and plasticity are maximized when the speed of environmental fluctuations are intermediate (Fig. 2*E*). Hence, plasticity is only adaptive when the timescale of environmental fluctuation is comparable to the timescales of the dynamics of the infection and of the social cue (*SI Appendix*, Fig. S4). In other words, we only expect plasticity to evolve when the temporal covariance can build up and this depends on i) parameters governing the spread of the pathogen, ii) parameters governing the spread of the social cue and iii) the speed of the environmental fluctuations. Although our model introduces temporal variation through time-dependent host influx, a covariance between the social cue and the availability of susceptible hosts is expected to arise if other ecological parameters, such as host reproduction or mortality rates, fluctuate over time. Hence, the fluctuation of other ecological factors are also expected to promote the evolution of pathogen plasticity.

Crucially an analysis of the spatially structured model indicates that covariance between the social cue and host availability can emerge even in the absence of externally imposed fluctuations of the environment. In a spatial lattice, the covariance arises endogenously from local and asynchronous outbreaks. These outbreaks deplete susceptible hosts locally, leading to pathogen fade-out and subsequent recolonization by susceptible hosts. This yields temporal variation in the dynamics of the infection and the information at the scale of a local epidemic. In fact we can show that the spatial covariance that appears in Eq. **5** is equivalent to a temporal (but local) covariance (*SI Appendix*, Fig. S2). This is an illustration of the “space-for-time” principle that emerges from the ergodicity of the dynamics on the spatial lattice: the pattern observed over time at a single location is equivalent to the pattern across space at a single point in time (52–55). The numerical exploration of a more complex ecological scenario with both externally imposed fluctuations and spatial structure showed the robustness of our results (*SI Appendix*, Fig. S7). Yet, the exploration of a broader range of scenarios combining spatial and temporal variation remains to be carried out.

Our study has important connections with several established theoretical frameworks. First, our model predicts that the evolution of plasticity is driven by the reliability of the cue used by the pathogen, which is captured by a covariance between the informed state and the quality of the environment. This is reminiscent of previous studies that have shown that plasticity is driven by the covariance between the environment experienced during the development of the individual and the environment experienced during selection (25, 27, 49, 56–58). While our life cycle assumptions do not allow us to distinguish between development and selection, a crucial distinction with these other studies is that the cue is not externally imposed but emerges from the demographic process through the density-dependent force of information Ω. This allows us to take into account the feedback loop between environmental dynamics and the reliability of the cue, and its effect on the evolution of plasticity. Second, our theoretical analysis has interesting conceptual links with previous studies that have explored connections between information theory and evolution (59–61). Indeed, the social cues released by infected individuals function as an information-gathering mechanism through which pathogens can evaluate transmission opportunities (39, 62, 63). The quality of this evaluation depends on the covariance of the cue with host availability. It is interesting that the direction of selection depends only on this covariance (a linear evaluation of the statistical association between these two quantities), and not on more complex measures of information like the “mutual information” used in information theory (59). Similarly, classical evolutionary theory based on the Price equation shows that the direction of selection of the mean phenotypic value depends only on the linear association (i.e., a covariance) between the phenotype and fitness (64–66). Third, it is worth noting that our evolutionary analysis has interesting parallels with theoretical developments in ecology, such as modern coexistence theory, which emphasize how spatiotemporal covariances can influence population growth rates and species coexistence through spatial storage or inflationary effects (67–69). As in our model, these effects are captured by covariances between the frequency of individuals in a given environmental state (e.g a patch or, as in our model, an information state) and the growth rates (of a species in ecology, or of a rare mutant in our evolutionary analysis).

An important empirical motivation of our study comes from communication systems in temperate phages [such as Arbitrium (31)]. While previous theoretical studies have shown that temporally variable environments can promote the evolution of plastic strategies mediated by the density of the Arbitrium signal (33, 38, 39), our analysis moves away from the specifics of the life cycle of temperate phages to develop a “minimal model” that retains the key factors driving the evolution of plasticity: the covariation between information and infection dynamics. This minimal model allows us to recover the effect of temporal fluctuations discussed in previous studies, but also to capture the effect of spatial structure. This effect is particularly relevant for the evolution of Arbitrium because temperate *Bacillus* phages that use Arbitrium communication live in soil environments where quorum sensing operates over short ranges (31, 32, 70). Next, it would be particularly interesting to study how other parameters of the model could coevolve with the plasticity of the pathogen. For instance, it would be particularly interesting to study how communication (parameters ω and γ) coevolves with plasticity. Interestingly, distantly related phages can produce similar, or even identical, Arbitrium peptides, leading to cross-talk that alters each other’s lysis–lysogeny decisions (71, 72). Such signal interference can generate social conflicts among different viruses and foster diverse types of interactions, including manipulation, cheating, and cooperation (36, 73).

Beyond Arbitrium, our framework can be extended to a broader range of biological systems. First, it is interesting to note that human malaria parasites (*Plasmodium falciparum*) have been shown to communicate within infected hosts via the production of microvesicles produced by malaria-infected erythrocytes (37). A higher density of microvesicles can stimulate the production of gametocytes (the transmission stage of malaria) and modulate a key step of the parasite development. The production of microvesicles can thus be viewed as a social signal used to coordinate the within-host exploitation of the population of erythrocytes. This shows the potential relevance of our work to understand the within-host dynamics of a human pathogen. Second, many hosts are known to use social cues to regulate defensive strategies against pathogens. For example, some bacteria employ quorum sensing to adjust CRISPR-Cas immunity or receptor expression (74–76), and some phages encode inhibitors of quorum sensing, potentially to reduce host defense expression (77). Besides, some social cues can be exchanged between hosts and pathogens, allowing each to influence the other’s life-history strategies. Certain phages can sense host-related signals–such as quorum sensing molecules, multiple infections, SOS responses, or the host’s metabolic state–and adjust their lysis–lysogeny decisions accordingly (78–84). In turn, bacteria can sense infection-linked “danger signals” released by nearby infected or lysed cells (e.g., peptidoglycan fragments) and upregulate defenses in advance, lowering susceptibility before they are directly infected (85, 86). The production of distinct social cues by the host and the pathogen may generate new evolutionary conflicts and drive the coevolution of life-history traits in both partners.

In order to capture the essential factors driving the evolution of pathogen plasticity, our model makes some simplifying assumptions. For instance, we assume that infected hosts cannot recover. It is clear that the presence of several immunity classes could affect the selective pressures on plasticity. Although a full treatment is beyond the scope of this study, it is interesting to note that, in a SIR model where infected hosts can recover and become fully immune to the disease, the selection gradients on class-specific virulence will take exactly the same form as in our work (*SI Appendix*, section 5). Thus, the qualitative prediction that plasticity is driven by the covariances $C_{1}^{\text{T}}$ and $C_{1}^{\text{S}}$ remains valid, although recovery could lead to quantitatively different predictions for the ESS virulence [as already found for spatial models without plasticity (14)]. In addition, the selection gradients in our work are derived using deterministic models, effectively assuming large population sizes. This suggests that fluctuations due to demographic stochasticity are not needed for plasticity to evolve. However, because we know that demographic stochasticity can affect the evolution of pathogen populations (87, 88), we have checked using individual-based simulations that our predictions are robust to a change in population size (*SI Appendix*, Fig. S8). Finally, we assume for simplicity that the perception of a social cue is binary (i.e., pathogens either detect the cue or not) and that the transmission and reception of the social cue are cost-free. In natural systems, however, responses to a cue are often dose-dependent and may incur metabolic or regulatory costs related to production, maintenance, or detection (89, 90). Our work can thus be viewed as a step toward a more general theoretical framework offering insights into the evolution of microbial “decision-making” in diverse ecological contexts.

## Supplementary Material

Appendix 01 (PDF)

## Data Availability

Numerical code data have been deposited in Zenodo (https://doi.org/10.5281/zenodo.20271193) (91).
