# Supplementary material for: Social cues drive the evolution of plastic pathogen virulence in spatially and temporally variable environments
Source: Proc Natl Acad Sci U S A. 2026 Jun 2;123(23):e2526952123. doi: 10.1073/pnas.2526952123 (PMC13250584; doi:10.1073/pnas.2526952123)
Supplement: Supplementary file 1 [file pnas.2526952123.sapp.pdf]

Supporting Information for

Social cues drive the evolution of plastic pathogen virulence in spatially and temporally variable environments

Ryuichi Kumata, Sébastien Lion, Edze Westra, Sylvain Gandon

Ryuichi Kumata
E-mail: ryuichi.kumata@cefe.cnrs.fr

This PDF file includes:

Supporting text
Figs. S1 to S9
SI References

Supporting Information Text

In this Supplementary Information we detail the derivation of the theoretical results presented in the main text. We contrast the effects of two different types of environmental variability (i.e., temporal and spatial) on the evolution of class-specific virulence. We first recall the general method to derive the selection gradient in class-structured populations in a constant or temporally fluctuating environment. Next we use this approach to investigate the evolution of plastic virulence in (i) a well-mixed model with no environmental variability, (ii) a model with temporal variability in external host influx, and (iii) a spatially structured model.

1. General approach for selection gradient in class-structured population

We consider a population structured in an arbitrary number of classes K. Let $\mathbf{n} = (n_1 \dots n_K)^\top$ be the vector of class densities and $\mathbf{M}$ the matrix of between-class transition rates. The dynamics of $\mathbf{n}$ take the form:

$$\frac{d\mathbf{n}}{dt} = \mathbf{M}\mathbf{n} \quad [1]$$

The dynamics of the total density $n = \mathbf{1}^\top \mathbf{n} = \sum_{k=1}^{K} n_k$ is:

$$\frac{dn}{dt} = \mathbf{1}^\top \mathbf{M}\mathbf{n} = \underbrace{\mathbf{1}^\top \mathbf{M}\mathbf{f}}_{\lambda} n \quad [2]$$

where $\mathbf{f} = \mathbf{n}/n$ is the vector of class frequencies and $\lambda = \mathbf{1}^\top \mathbf{M}\mathbf{f}$ is the per-capita growth rate of the population. Combining these two equations yields:

$$\frac{d\mathbf{f}}{dt} = \mathbf{M}\mathbf{f} - \lambda \mathbf{f} \quad [3]$$

At an ecological equilibrium, the per-capita growth rate λ is zero and the latter equation reduces to

$$\mathbf{M}\mathbf{f} = \mathbf{0} \quad [4]$$

so the stable class distribution $\mathbf{f}$ is a right eigenvector associated to eigenvalue $\lambda = 0$ (1–3). We similarly define the vector of individual reproductive values in each class as a left eigenvector associated to eigenvalue $\lambda = 0$ (1–3):

$$\mathbf{v}^\top \mathbf{M} = \mathbf{0}^\top \quad [5]$$

Invasion dynamics: Consider the invasion of the rare mutant into a resident population at its ecological equilibrium. We note $\mathbf{M}^m$ the invasion matrix obtained from linearizing the dynamics of the rare mutant near the resident equilibrium. Then, the densities of mutant, $\mathbf{n}^m(t)$, change asymptotically (1, 2) as

$$\mathbf{n}^m(t) \sim c e^{\lambda^m t} \mathbf{f}^m \quad [6]$$

where λ^m is the dominant eigenvalue of $\mathbf{M}^m$ and $\mathbf{f}^m$ is the associated right eigenvector normalized as $\mathbf{1}^\top \mathbf{f}^m = 1$ (stable class distribution). This states that the population of the rare mutant grows exponentially while maintaining a stable class distribution. In other words, at the stable class distribution, the growth rate in each class is the same as the growth rate of the total population. For instance if we have two classes A and B, we have

$$\lambda^m = \mathbf{1}^\top \mathbf{M}^m \mathbf{f}^m = \frac{1}{n_A}\frac{dn_A}{dt} = \frac{1}{n_B}\frac{dn_B}{dt} = \frac{1}{n}\frac{dn}{dt} \quad [7]$$

By definition

$$\mathbf{M}^m \mathbf{f}^m = \lambda^m \mathbf{f}^m \quad [8]$$

Let $\mathbf{w}$ be the arbitrary vector of weights such that $\mathbf{w}^\top \mathbf{f}^m = 1$. Then, we have:

$$\mathbf{w}^\top \mathbf{M}^m \mathbf{f}^m = \mathbf{w}^\top \lambda^m \mathbf{f}^m = \lambda^m \quad [9]$$

Selection gradient: Under weak selection, we can write down the following expansion of Eq. (9) with :

$$\lambda^m = (\mathbf{w}^\top + \varepsilon\partial\mathbf{w}^\top)(\mathbf{M} + \varepsilon\partial\mathbf{M})(\mathbf{f} + \varepsilon\partial\mathbf{f}) + O(\varepsilon^2) \quad [10]$$

$$= \mathbf{w}^\top\mathbf{M}\mathbf{f} + \varepsilon\partial\mathbf{w}^\top\mathbf{M}\mathbf{f} + \varepsilon\mathbf{w}^\top\partial\mathbf{M}\mathbf{f} + \varepsilon\mathbf{w}^\top\mathbf{M}\partial\mathbf{f} + O(\varepsilon^2) \quad [11]$$

At the ecological equilibrium, the resident dynamics is neutral ($\mathbf{M}\mathbf{f} = \lambda = 0$), then:

$$\lambda^m = \varepsilon(\mathbf{w}^\top\partial\mathbf{M}\mathbf{f} + \mathbf{w}^\top\mathbf{M}\partial\mathbf{f}) + O(\varepsilon^2) \quad [12]$$

This general selection gradient in a class-structured population can be used to obtain two different types of gradients(4–7).

First, one can use the following weight vector $\mathbf{w} = \mathbf{1}$, which yields (4):

$$\mathcal{S} = \mathbf{1}^\top\partial\mathbf{M}\mathbf{f} + \mathbf{1}^\top\mathbf{M}\partial\mathbf{f} \quad [13]$$

Second, one can use the following weight vector $\mathbf{w} = \mathbf{v}$, which is defined as the left eigenvector associated with the eigenvalue $\lambda = 0$ of $\mathbf{M}$ (5). We can normalise these reproductive values so that $\mathbf{v}^\top\mathbf{f} = 1$. Then, the selection gradient becomes:

$$\mathcal{S} = \mathbf{v}^\top\partial\mathbf{M}\mathbf{f} \quad [14]$$

Despite having different expressions, both selection gradients give the same prediction for the direction of selection. We derive both gradients of selection below but we focus on the one without reproductive values (Eq. (13)) in the main text because these reproductive values are difficult to compute in the spatial model. We refer to the gradient in Eq. (13) as the frequency-based gradient, and that in Eq. (14) as the RV-based gradient.

Temporally fluctuating environment: The above approach can be extended to the temporally fluctuating population (8). From the dynamics of $\mathbf{n}$

$$\frac{d\mathbf{n}}{dt} = \mathbf{M}(t)\mathbf{n}(t) \quad [15]$$

we can calculate the instantaneous per-capita growth rate of the resident is

$$\lambda(t) = \mathbf{1}^\top\mathbf{M}(t)\mathbf{f}(t) \quad [16]$$

From this it follows that the dynamics of the class frequency $\mathbf{f}(t)$ is given by

$$\frac{d\mathbf{f}}{dt} = \mathbf{M}(t)\mathbf{f}(t) - \lambda(t)\mathbf{f}(t). \quad [17]$$

Similarly, the reproductive values $\mathbf{v}(t)$ become a dynamical variable with the following dynamics:

$$\frac{d\mathbf{v}^\top}{dt} = -\mathbf{v}^\top(t)\mathbf{M}(t) + \lambda(t)\mathbf{v}^\top(t), \quad [18]$$

along with the normalization $\mathbf{v}^\top(t)\mathbf{f}(t) = 1$. These two equations generalises to time-varying environments the classical definitions of $\mathbf{f}$ (resp. $\mathbf{v}$) as a right (resp. left) eigenvector of the matrix M(t) (6, 7). Importantly, $\mathbf{v}^\top(t)\mathbf{f}(t)$ is normalised to one at all time t if $\mathbf{v}^\top(t_e)\mathbf{f}(t_e) = 1$ is satisfied at time t_e because $\mathbf{v}^\top(t)\mathbf{f}(t)$ is constant:

$$\frac{d}{dt}\left(\mathbf{v}^\top(t)\mathbf{f}(t)\right) = \frac{d\mathbf{v}^\top}{dt}\mathbf{f}(t) + \mathbf{v}(t)^\top\frac{d\mathbf{f}}{dt} = 0.$$

Now consider a rare mutant invasion into the periodical resident attractor. Denoting by $\lambda^m(t)$ the instantaneous rate of the invading mutant, we have

$$\lambda^m(t) = \mathbf{1}^\top\mathbf{M}^m(t)\mathbf{f}^m(t) \quad [19]$$

where $\mathbf{M}^m(t)$ and $\mathbf{f}^m(t)$ are the instantaneous mutant invasion matrix and class frequency distribution at a given time t, respectively. The invasion fitness of the mutant is determined by the average of the per-capita growth rate $\lambda^m(t)$ over one period of the resident attractor (8–10). Under weak selection ($\mathbf{M}^m(t) = \mathbf{M}(t) + \epsilon\partial\mathbf{M}(t) + o(\epsilon^2)$), the instantaneous selection gradient can be written in two equivalent forms:

- Frequency-based:

$$\mathcal{S}(t) = \mathbf{1}^\top\partial\mathbf{M}(t)\mathbf{f}(t) + \mathbf{1}^\top\mathbf{M}(t)\partial\mathbf{f}(t) \quad [20]$$

- Reproductive-value(RV)-based:

$$\mathcal{S}(t) = \mathbf{v}(t)^\top\partial\mathbf{M}(t)\mathbf{f}(t) \quad [21]$$

The long-term direction of selection can be predicted by averaging the instantaneous selection gradient over one period of the fluctuation (8).

2. Constant and well-mixed model

The model describes the transmission of the pathogen and the transmission of information among hosts in a constant, well-mixed environment (Fig. 1). This yields the following system of ordinary differential equations (where the dependence on t of dynamical variables is dropped to simplify the notation):

$$\begin{aligned}\frac{dS_0}{dt} &= \theta + rS(1-N) - (\beta_0 I_0 + \beta_1 I_1)S_0 - \omega I S_0 - dS_0 + \gamma S_1\\ \frac{dS_1}{dt} &= -(\beta_0 I_0 + \beta_1 I_1)S_1 + \omega I S_0 - dS_1 - \gamma S_1\\ \frac{dI_0}{dt} &= (\beta_0 I_0 + \beta_1 I_1)S_0 - \omega I I_0 - (d+\alpha_0)I_0 + \gamma I_1\\ \frac{dI_1}{dt} &= (\beta_0 I_0 + \beta_1 I_1)S_1 + \omega I I_0 - (d+\alpha_1)I_1 - \gamma I_1\end{aligned} \quad [22]$$

Epidemiological dynamics and information dynamics can be separated. First, the total density of susceptible hosts $S = S_0+S_1$ and the total density of infected hosts $I = I_0 + I_1$, which yields:

$$\begin{aligned}\frac{dS}{dt} &= \theta + rS(1-N) - \bar{\beta} SI - dS\\ \frac{dI}{dt} &= \left(\bar{\beta}S - (d+\bar{\alpha})\right) I\end{aligned} \quad [23]$$

where $\bar{\beta} = (1-f_1)\beta_0 + f_1\beta_1$ is the average transmission rate over the class distribution and $\bar{\alpha} = \alpha_0(1-f_1) + \alpha_1 f_1$ is the average virulence. The frequency of informed individuals among susceptible hosts is noted $g_1 = S_1/S$ and the frequency of informed individuals among infected hosts is noted $f_1 = I_1/I$.

At the disease-free equilibrium there is no infection and the density of susceptible hosts is

$$S_\varnothing = \frac{1}{2r}\left(r - d + \sqrt{(r-d)^2 + 4r\theta}\right).$$

Second, the dynamics of information are described by:

$$\begin{aligned}\frac{dg_1}{dt} &= -\bar{\beta} I g_1 + \omega I(1-g_1) - \left(d + \gamma + r_w^S\right) g_1\\ \frac{df_1}{dt} &= \bar{\beta} S g_1 + \omega I(1-f_1) - (d + \alpha_1 + \gamma f_1 + r_w^I) f_1\end{aligned} \quad [24]$$

where $r_w^S = \theta/S + r(1-N) - \bar{\beta} I - d$ and $r_w^I = \bar{\beta} S - d - \bar{\alpha}$ are the per-capita growth rates of susceptible and infected hosts, respectively.

Extensive simulations indicate that the dynamics of this system converges to a single endemic equilibrium when the basic reproduction ratio $R_0 = \beta_0 S_\varnothing/(d+\alpha_0) > 1$. A rigorous demonstration of the global stability of this endemic equilibrium, however, remains to be carried out. Combining Eq. (23) and Eq. (24), the equilibrium densities of epidemiological dynamics can be expressed using the averaged epidemiological parameters

$$(\hat{S}, \hat{I}) = \left(\frac{d+\bar{\alpha}}{\bar{\beta}}, \frac{\bar{\beta}\theta_0}{(r+\bar{\beta})(d+\bar{\alpha})} - \frac{(d+\bar{\alpha})r + (d-r)\bar{\beta}}{\bar{\beta}(r+\bar{\beta})}\right) \quad [25]$$

and the equilibrium frequencies of the informed class are

$$(\hat{g}_1, \hat{f}_1) = \left(\frac{\omega \hat{I}}{\omega\hat{I} + \bar{\beta}\hat{I} + d + \gamma}, \frac{\bar{\beta}\hat{S}\hat{g}_1 + \omega\hat{I}}{\omega\hat{I} + d + \bar{\alpha} + \gamma}\right) \quad [26]$$

Because the averaged phenotypes ($\bar{\beta}$ and $\bar{\alpha}$) depend on the equilibrium frequency of the infected class $\hat{f}_1$, the above equilibrium densities and frequencies are only implicitly defined, but can be numerically calculated. The information free equilibrium ($\hat{g}_1 = \hat{f}_1 = 0$) is reached when no information spreads ($\omega = 0$) or there is no infected host ($\hat{I} = 0$). In other words, some fraction of hosts in the host population is informed as long as the pathogen is endemic and can transmit some information.

A. Evolution of virulence in a constant and well-mixed model. Before stepping into the evolution of the class-specific virulence, we consider the evolution of virulence under the constraint of non-plasticity ($\alpha_0 = \alpha_1 = \alpha$). In this case, the epidemiological dynamics follows Eq. (23) with $\bar{\beta} = \beta$ and $\bar{\alpha} = \alpha$. The invasion dynamics of the mutant with virulence α^m into the ecological equilibrium of the resident having with virulence α is

$$\frac{dI^m}{dt} = \Big[\beta(\alpha^m)\hat{S} - (d+\alpha^m)\Big] I^m = \lambda^m I^m, \quad [27]$$

where $\hat{S}$ is the equilibrium density of the susceptible host given in Eq. (25) and λ^m gives the invasion fitness of a rare mutant. The direction of selection under weak selection is given by the first-order effect of mutation on λ^m, which gives the selection gradient (1, 11)

$$\mathcal{S} = \beta'(\alpha)\hat{S} - 1. \quad [28]$$

In this study, we assume that the trade-off function $\beta(\alpha)$ between virulence and transmission is increasing and saturating ($\beta' > 0, \beta'' < 0$), as in classical models of virulence evolution (12). Under this trade-off, the singular trait α^* (defined by the vanishing selection gradient) is both evolutionarily stable and convergence stable (i.e. a continuously stable strategy (CSS) in Adaptive Dynamics).

Evolutionary stability Because the resident equilibrium $\hat{S}$ is fixed when differentiating with respect to the mutant trait α_m, we have

$$\left.\frac{\partial^2 \lambda^m}{\partial \alpha_m^2}\right|_{\alpha_m=\alpha^*} = \beta''(\alpha^*)\,\hat{S}(\alpha^*). \quad [29]$$

Since $\hat{S}(\alpha^*) > 0$, the ESS condition reduces to $\beta''(\alpha^*) < 0$, which is true by assumption.

Convergence stability. The derivative of the selection gradient is

$$\left.\frac{\partial}{\partial \alpha}\left(\left.\frac{\partial \lambda^m}{\partial \alpha_m}\right|_{\alpha_m=\alpha}\right)\right|_{\alpha=\alpha^*} = \beta''(\alpha^*)\hat{S}(\alpha^*) + \beta'(\alpha^*)\left.\frac{\partial \hat{S}(\alpha)}{\partial \alpha}\right|_{\alpha=\alpha^*}. \quad [30]$$

Using $\hat{S}(\alpha) = (d+\alpha)/\beta(\alpha)$ from Eq. (25), we obtain

$$\frac{\partial \hat{S}(\alpha)}{\partial \alpha} = \frac{\beta(\alpha) - (d+\alpha)\beta'(\alpha)}{\beta(\alpha)^2}, \quad [31]$$

which implies $\left.\frac{\partial \hat{S}(\alpha)}{\partial \alpha}\right|_{\alpha=\alpha^*} = 0$ because the singular condition is $\beta'(\alpha^*) = \beta(\alpha^*)/(d+\alpha^*)$. Hence

$$\left.\frac{\partial}{\partial \alpha}\left(\left.\frac{\partial \lambda^m}{\partial \alpha_m}\right|_{\alpha_m=\alpha}\right)\right|_{\alpha=\alpha^*} = \beta''(\alpha^*)\hat{S}(\alpha^*) < 0, \quad [32]$$

so α^* is convergence stable as well.

From the gradient (Eq. (28)), we find that the slope of the tradeoff function, $\beta(\alpha)$, at the ESS level of virulence α^* is equal to the inverse of the equilibrium density of susceptibles $\hat{S}$:

$$\beta'(\alpha^*) = \frac{1}{\hat{S}} \quad [33]$$

This clearly shows the ESS level of virulence is linked to the availability of the susceptible hosts. If the host availability is small, the $\beta'(\alpha^*)$ becomes large, which means the α^* is small under the assumed trade-off. This link is useful for interpretation of the class-specific virulence evolution in the following different scenarios (temporally fluctuating population and spatially structured population).

With the equilibrium value of susceptible hosts, $\hat{S} = (d+\alpha)/\beta$ (Eq. (25)), the ESS values of virulence α^* must satisfy

$$\beta'(\alpha^*) = \frac{\beta}{d+\alpha^*} \quad [34]$$

which is a classical result of virulence evolution theory, in the form of a marginal value theorem. In this study, we use the tradeoff between transmission and virulence $\beta(\alpha) = \beta_{max}\alpha^{1/\kappa}$. Under the tradeoff, the ESS virulence is

$$\alpha^* = \frac{d}{\kappa - 1} \quad [35]$$

In the following we focus on the scenario where $\kappa = 2$ which yields the ESS virulence $\alpha^* = d$.

Note that the equilibrium density of susceptibles $\hat{S}$ depends on the level of virulence (see Eq. (25)) and this link drives the ecological and evolutionary feedback. In this simple scenario of virulence evolution, the pathogen evolves to achieve the lowest density of susceptibles, which is the worst scenario for the pathogen (pessimisation principle) (13, 14).

B. Evolution of class-specific virulence in a constant and well-mixed model. The dynamics of rare mutant with virulence (α_0^m, α_1^m) when the resident viruses with (α_0, α_1) is at equilibrium:

$$\frac{\mathrm{d}}{\mathrm{d}t}\begin{pmatrix} I_0^m \\ I_1^m \end{pmatrix} = \mathbf{M}^m \begin{pmatrix} I_0^m \\ I_1^m \end{pmatrix}$$

with an invasion matrix of the mutant

$$\mathbf{M}^m = \begin{pmatrix} \beta(\alpha_0^m)\hat{S}_0 - (d+\alpha_0^m) - \omega I & \beta(\alpha_1^m)\hat{S}_0 + \gamma \\ \beta(\alpha_0^m)\hat{S}_1 + \omega I & \beta(\alpha_1^m)\hat{S}_1 - (d+\alpha_1^m+\gamma) \end{pmatrix}. \quad [36]$$

In the same way, the dynamics of the resident with (α_0, α_1) is given by

$$\frac{\mathrm{d}}{\mathrm{d}t}\begin{pmatrix} I_0 \\ I_1 \end{pmatrix} = \mathbf{M} \begin{pmatrix} I_0 \\ I_1 \end{pmatrix}$$

with the resident matrix $\mathbf{M}$

$$\mathbf{M} = \begin{pmatrix} \beta(\alpha_0)\hat{S}_0 - (d+\alpha_0) - \omega \hat{I} & \beta(\alpha_1)\hat{S}_0 + \gamma \\ \beta(\alpha_0)\hat{S}_1 + \omega \hat{I} & \beta(\alpha_1)\hat{S}_1 - (d+\alpha_1+\gamma) \end{pmatrix}$$

Following Eq. (13), the selection gradient for class specific virulence, α_i, is

$$\mathcal{S}_i = f_i\left(\beta'(\alpha_i)\hat{S} - 1\right) + \Delta\rho\frac{\partial f_1^m}{\partial \alpha_i^m} \quad [37]$$

where $\beta'(\alpha_i)$ denotes the derivative of $\beta(\alpha)$ by α evaluated at $\alpha_i^m = \alpha_i$ and $\Delta\rho = \rho_1 - \rho_0$ and ρ_i is the growth rate of the population in each class in the absence of class transition, $\rho_i = \beta_i S - (d + \alpha_i)$. The second term of Eq. (37) describes the indirect effect of mutations on selection via the modification of the frequency of informed hosts among the hosts infected by the mutant. Following Eq. (14), we obtain an alternative expression for the selection gradient based on reproductive values for class specific virulence as

$$\mathcal{S}_i = f_i\left(\beta'(\alpha_i)\hat{S}_{\mathbf{v}} - v_i\right) \quad [38]$$

where $\hat{S}_{\mathbf{v}} = \hat{S}_0 v_0 + \hat{S}_1 v_1$ is the effective total density of susceptible hosts, weighted by the individual reproductive values.

Non-plastic situation: In the non-plastic situation (i.e. $\alpha_0 = \alpha_1 = \alpha$),

$$\Delta\rho = 0$$

since $\rho_0 = \rho_1 = \beta\hat{S} - (d+\alpha)$. Plugging this into eq. Eq. (37), the second term vanishes and the selection gradient of class specific virulence becomes

$$\mathcal{S}_i = f_i\left(\beta'(\alpha)\hat{S} - 1\right) \quad [39]$$

Importantly, the selection gradient for class-specific virulence at the non-plastic ESS ($\alpha_0 = \alpha_1 = \alpha^*$) vanishes with the non-plastic ESS condition in Eq. (33):

$$\mathcal{S}_i = f_i(\beta'(\alpha^*)\hat{S} - 1) = 0 \quad [40]$$

which means there is no selection for plasticity when we start from the no-plastic ESS virulence.

From the class-specific selection gradient (Eq. (39)), we can numerically confirm that selection always drives virulence towards the non-plastic ESS virulence α^* (Fig. S1). It is important to note, however, that pathogens can be plastic *transiently* while evolving toward the non-plastic ESS. Even when starting from non-plastic virulence value different from α^*, selection can temporarily generate plasticity. The reason is that Eq. (39) is weighted by class frequencies, which creates asymmetric selection between α_0 and α_1 . Indeed, when the information spreads slowly (A), the frequency of informed class tends to be low and α_0 evolves first, and α_1 follows more gradually. In contrast, when information spreads rapidly (B), the frequency of informed class goes higher and α_1 evolves first, and α_0 then changes more gradually.

Joint ESS of a constant and well-mixed model: Under the assumed condition on the trade-off ($\beta' > 0, \beta'' < 0$), it can be proved that $(\alpha_0, \alpha_1) = (\alpha^*, \alpha^*)$ is the unique joint ESS,

Proposition. *The non-plastic ESS, α^*, is the unique joint ESS in a constant and well-mixed model.*

Proof. Assume for contradiction that there exists a plastic joint ESS $(\alpha_0^\dagger, \alpha_1^\dagger)$ with $\alpha_0^\dagger \neq \alpha_1^\dagger$. Let $\hat{S}$ be the total susceptible density at the resident endemic equilibrium. From Eq. (23),

$$\hat{S} = \frac{d+\bar{\alpha}}{\bar{\beta}}, \qquad \bar{\alpha} = (1-\hat{f}_1)\alpha_0^\dagger + \hat{f}_1\alpha_1^\dagger, \quad \bar{\beta} = (1-\hat{f}_1)\beta(\alpha_0^\dagger) + \hat{f}_1\beta(\alpha_1^\dagger).$$

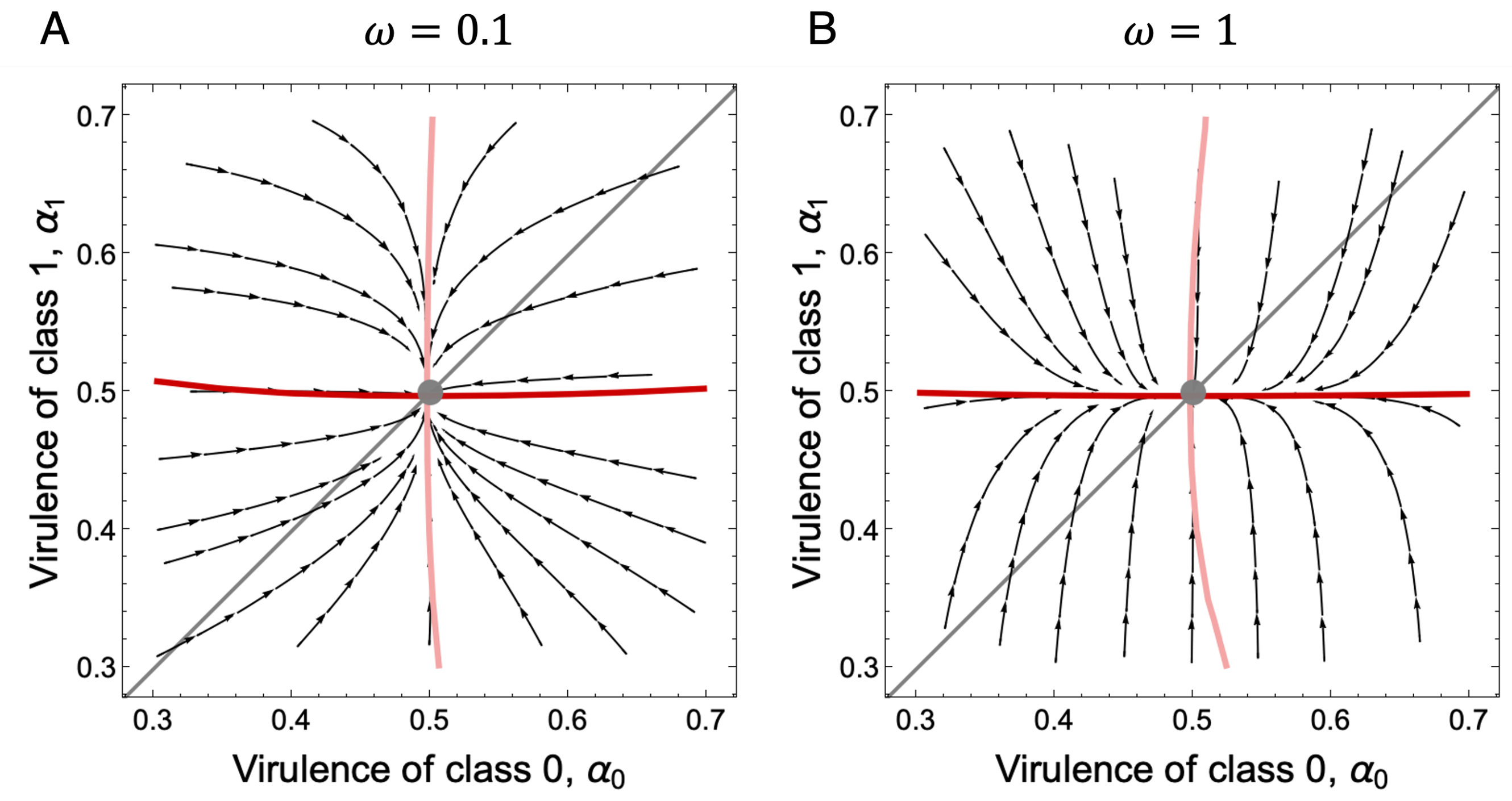

Fig. S1. Selection toward non-plastic ESS in the well-mixed and constant population. Each streamline shows the direction of selection when $\omega = 0.1$ (A) and $\omega = 1$ (B). Pink and red curves indicate the evolutionary nullcline of α_0 and α_1, respectively. Other parameter values: $\theta_A = 10, \theta_B = 1, \beta_{max} = 5, \omega = 1, \gamma = 1, r = 1, d = 0.5$.

Consider a non-plastic mutant with $\alpha_0^m = \alpha_1^m = \bar{\alpha}$. Its per-capita invasion growth rate in the resident environment is

$$\lambda_m = \beta(\bar{\alpha})\hat{S} - (d + \bar{\alpha}) = (d + \bar{\alpha})\left(\frac{\beta(\bar{\alpha})}{\bar{\beta}} - 1\right).$$

Since β is strictly concave ($\beta'' < 0$), Jensen's inequality yields $\beta(\bar{\alpha}) \geq \bar{\beta}$, with strict inequality when $\hat{f}_1 \in (0, 1)$ and $\alpha_0^\dagger \neq \alpha_1^\dagger$. Hence $\lambda_m > 0$, and the mutant invades, implying that $(\alpha_0^\dagger, \alpha_1^\dagger)$ cannot be a joint ESS. Therefore any joint ESS must be non-plastic: $\alpha_0^\dagger = \alpha_1^\dagger = \alpha^*$.

□

3. Temporally fluctuating model

In this section, we assume that the host influx $\theta(t)$ is periodically fluctuating in time. Extensive simulations indicate that the dynamics of this system converges to a single periodic attractor when the basic reproduction ratio of the pathogen is higher than 1*. A rigorous demonstration of the global stability of this periodic attractor, however, remains to be carried out.

A. Evolution of class specific virulence with temporally fluctuations. Following the approach described in section S1, we can derive the selection gradient in a temporally fluctuating population. The invasion dynamics of the mutant with (α_0^m, α_1^m) are

$$\frac{\mathrm{d}}{\mathrm{d}t}\begin{pmatrix} I_0^m \\ I_1^m \end{pmatrix} = \mathbf{M}^m(t)\begin{pmatrix} I_0^m \\ I_1^m \end{pmatrix}$$

with a temporally fluctuating invasion matrix

$$\mathbf{M}^m(t) = \begin{pmatrix} \beta(\alpha_0^m)S_0(t) - (d+\alpha_0^m) - \omega I(t) & \beta(\alpha_1^m)S_0(t) + \gamma \\ \beta(\alpha_0^m)S_1(t) + \omega I(t) & \beta(\alpha_1^m)S_1(t) - (d + \alpha_1^m + \gamma) \end{pmatrix}. \quad [41]$$

Because we want to characterise the invasion of the mutant when the resident population has reached an equilibrium the densities of the different types of hosts have to be computed at this equilibrium. In a periodically fluctuating scenario this equilibrium is expected to be a periodic attractor. In the following, all the epidemiological variables $(S_0(t), S_1(t)...)$ are implicitly calculated on the periodic attractor.

In the same way, the dynamics of the resident wild-type with (α_0, α_1) is given by the resident matrix

$$\mathbf{M}(t) = \begin{pmatrix} \beta(\alpha_0)S_0(t) - (d+\alpha_0) - \omega I(t) & \beta(\alpha_1)S_0(t) + \gamma \\ \beta(\alpha_0)S_1(t) + \omega I(t) & \beta(\alpha_1)S_1(t) - (d + \alpha_1 + \gamma) \end{pmatrix} \quad [42]$$

Using Eq. (17) and Eq. (18), the resident matrix $\mathbf{M}(t)$ yields the dynamics of class frequencies as

$$\frac{df_0}{dt} = (\rho_0(t) - \omega I - \lambda(t))f_0 + (\beta(\alpha_1)S_0(t) + \gamma)f_1 \quad [43]$$

$$\frac{df_1}{dt} = (\beta(\alpha_0)S_1(t) + \omega I(t))f_0 + (\rho_1(t) - \gamma - \lambda(t))f_1 \quad [44]$$

and the dynamics of reproductive values as

$$\frac{dv_0}{dt} = (\omega I(t) - \rho_0(t))v_0(t) - (\beta(\alpha_0)S_1(t) + \omega I(t))v_1(t) + \lambda(t)v_0(t) \quad [45]$$

$$\frac{dv_1}{dt} = (\gamma - \rho_1(t))v_1(t) - (\beta(\alpha_1)S_0(t) + \gamma)v_0(t) + \lambda(t)v_1(t) \quad [46]$$

where $\rho_i(t) = \beta(\alpha_i)S_i(t) - (d + \alpha_i)$.

Frequency-based selection gradient: Using Eq. (20), the instantaneous selection gradient for class-specific virulence is given by

$$\mathcal{S}_i(t) = f_i(t)\left(S(t)\beta'(\alpha_i) - 1\right) + \Delta\rho\frac{\partial f_1^m}{\partial \alpha_i^m} \quad [47]$$

and its temporal average yields the selection gradient given in the main text (equation (5)) as a sum of three components ($\mathcal{T}_i^{\mathrm{T}}$, $\mathcal{F}_i^{\mathrm{T}}$ and $\mathcal{C}_i^{\mathrm{T}}$):

$$\mathcal{S}_i^{\mathrm{T}} = \langle \mathcal{S}_i(t)\rangle = \underbrace{\langle f_i\rangle\left(\beta'(\alpha_i)\langle S\rangle - 1\right)}_{\mathcal{T}_i^{\mathrm{T}}} + \underbrace{\left\langle \Delta\rho\frac{\partial f_1^m}{\partial \alpha_i^m}\right\rangle}_{\mathcal{F}_i^{\mathrm{T}}} + \underbrace{\beta'(\alpha_i)\mathrm{Cov}^{\mathrm{T}}(f_i, S)}_{\mathcal{C}_i^{\mathrm{T}}} \quad [48]$$

where $\langle\cdot\rangle$ means taking the arithmetic average over one period and $\mathrm{Cov}^T(f_i, S)$ is the temporal covariance, which is defined by

$$\mathrm{Cov}^{\mathrm{T}}(f_i, S) = \langle f_i S\rangle - \langle f_i\rangle\langle S\rangle \quad [49]$$

Note that $\mathrm{Cov}^{\mathrm{T}}(f_0, S) = -\mathrm{Cov}^{\mathrm{T}}(f_1, S)$ due to the conservation of probability $(f_0 + f_1 = 1)$ and the linearity of covariance.

RV-based selection gradient: Following Eq. (21), we obtain the alternative expression for the selection gradient for class specific virulence in the temporally fluctuating population as

$$\mathcal{S}_i^{\mathrm{T}} = \langle f_i\rangle\beta'(\alpha_i)\langle S_{\mathbf{v}}\rangle - \langle f_i\, v_i\rangle + \beta'(\alpha_i)\mathrm{Cov}^{\mathrm{T}}(f_i, S_{\mathbf{v}}) \quad [50]$$

where $S_{\mathbf{v}} = S_0 v_0 + S_1 v_1$ is the effective density of susceptible hosts, weighted by reproductive values. Note that there is no equivalent of the $\mathcal{F}_i^T$ term in this case. In order to confirm that the two gradients Eq. (48) and Eq. (50) give the same prediction, we show in Fig. S2 that they yield the same evolutionary trajectory when we start from the non-plastic ESS.

*Note that the derivation of R_0 is more challenging in a time-varying environment (15–17)

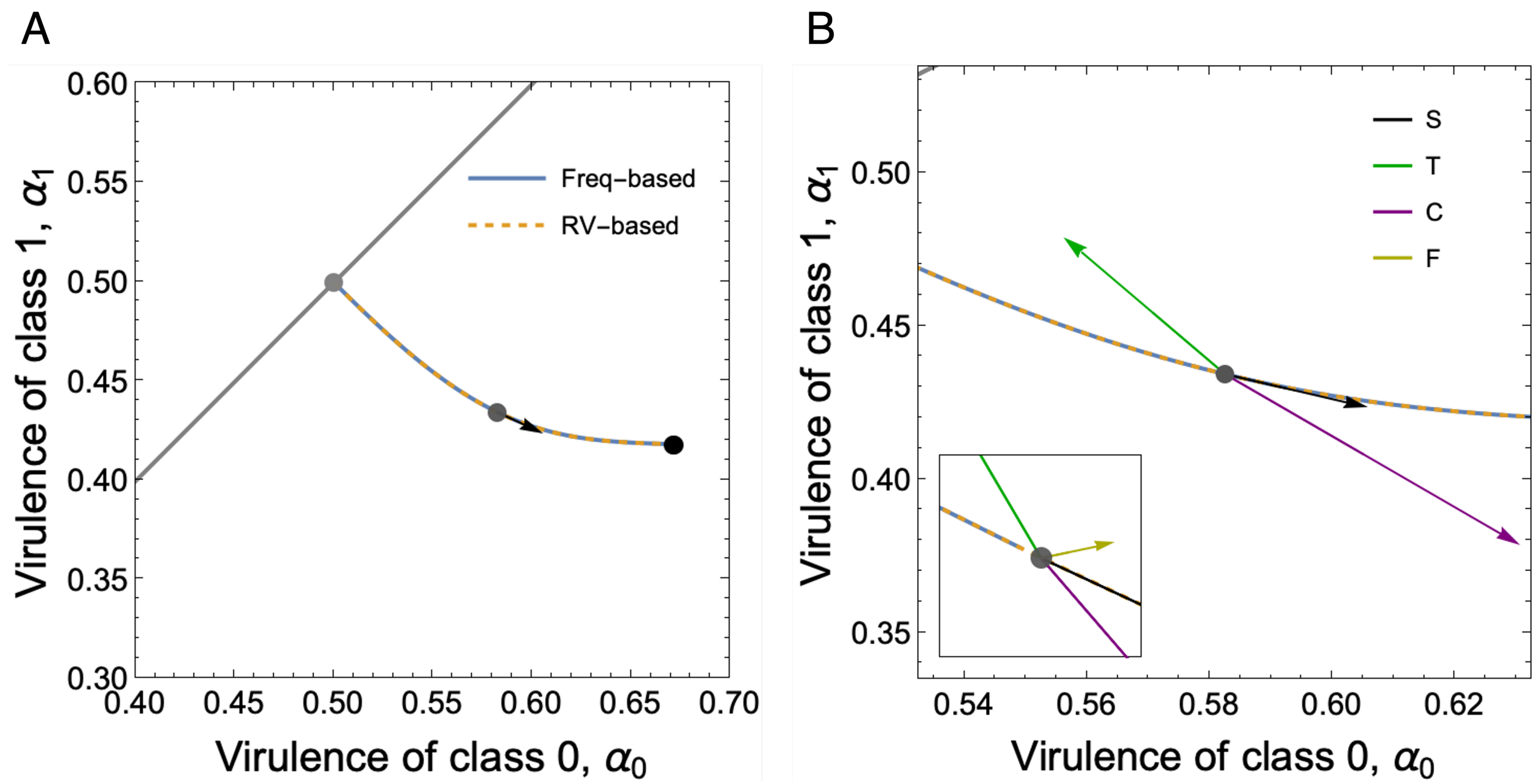

Fig. S2. Geometrical composition of the selection gradient. (A) The evolutionary trajectories predicted by the selection gradient in freq-based (solid, blue) and in RV-based (dashed, orange). The grey line indicates non-plastic strategies and the gray point is the non-plastic ESS. The black point is the joint ESS. The vector is the selection gradient at an intermediate point. (B) Vectors of the selection gradient and its components at the intermediate point in A. The inset plot is an expanded view around the focal point, showing the component $\vec{\mathcal{F}}^{\mathrm{S}}$ is very small compared to other components. Other parameter values: $\theta_A = 10, \theta_B = 1, \beta_{max} = 5, \omega = 1, \gamma = 1, r = 1, d = 0.5$.

Non-plastic scenario: In the non-plastic case (i.e., $\beta_0 = \beta_1 = \beta, \alpha_0 = \alpha_1 = \alpha$), the above selection gradient for class-specific virulence can be reduced to a simpler expression. In the non-plastic case, the dynamics of resident parasite $I = I_0 + I_1$ is

$$\frac{dI}{dt} = (\beta S(t) - (d + \alpha))\, I \tag{51}$$

On the periodic attractor we have $\langle d \ln I/dt \rangle = 0$ and thus

$$\langle S \rangle = \frac{d + \alpha}{\beta} \tag{52}$$

The selection gradient for non-plastic virulence α is

$$\mathcal{S}^{\mathrm{T}} = \beta'(\alpha)\langle S \rangle - 1 \tag{53}$$

Notably, this gradient gives the same gradient of virulence in the constant and well-mixed model Eq. (28). We thus recover the classical ESS condition of the constant and well-mixed model

$$\beta'(\alpha^*) = \frac{\beta}{d + \alpha^*} \tag{54}$$

This means that non-plastic ESS virulence in the temporally fluctuating case is the ESS value α^* in the constant and well-mixed model. At the non-plastic ESS ($\alpha_0 = \alpha_1 = \alpha^*$), the first and second terms in the selection gradient vanishes. Then, the selection gradient is reduced to a simplified form

$$\mathcal{S}_i^{\mathrm{T}} = \beta'(\alpha^*)\mathrm{Cov}^{\mathrm{T}}(f_i, S) \tag{55}$$

B. Alternative shapes of temporal fluctuations. In the main text, we use a square wave function to model the temporal fluctuation in host influx. To check the robustness of our results on the assumption of the function form, we assume the following temporal fluctuation in the host influx with period $T = 2\pi/\nu$ (where ν is the angular frequency of the fluctuation)

$$\theta(t) = \theta_0 + \delta \cos(\nu t) \tag{56}$$

where the amplitude δ of the temporal fluctuation. Importantly, this fluctuations also shows the similar results in the main text (Compare Fig. S3 with Fig. 2), suggesting the robustness of our results on the shape of fluctuation.

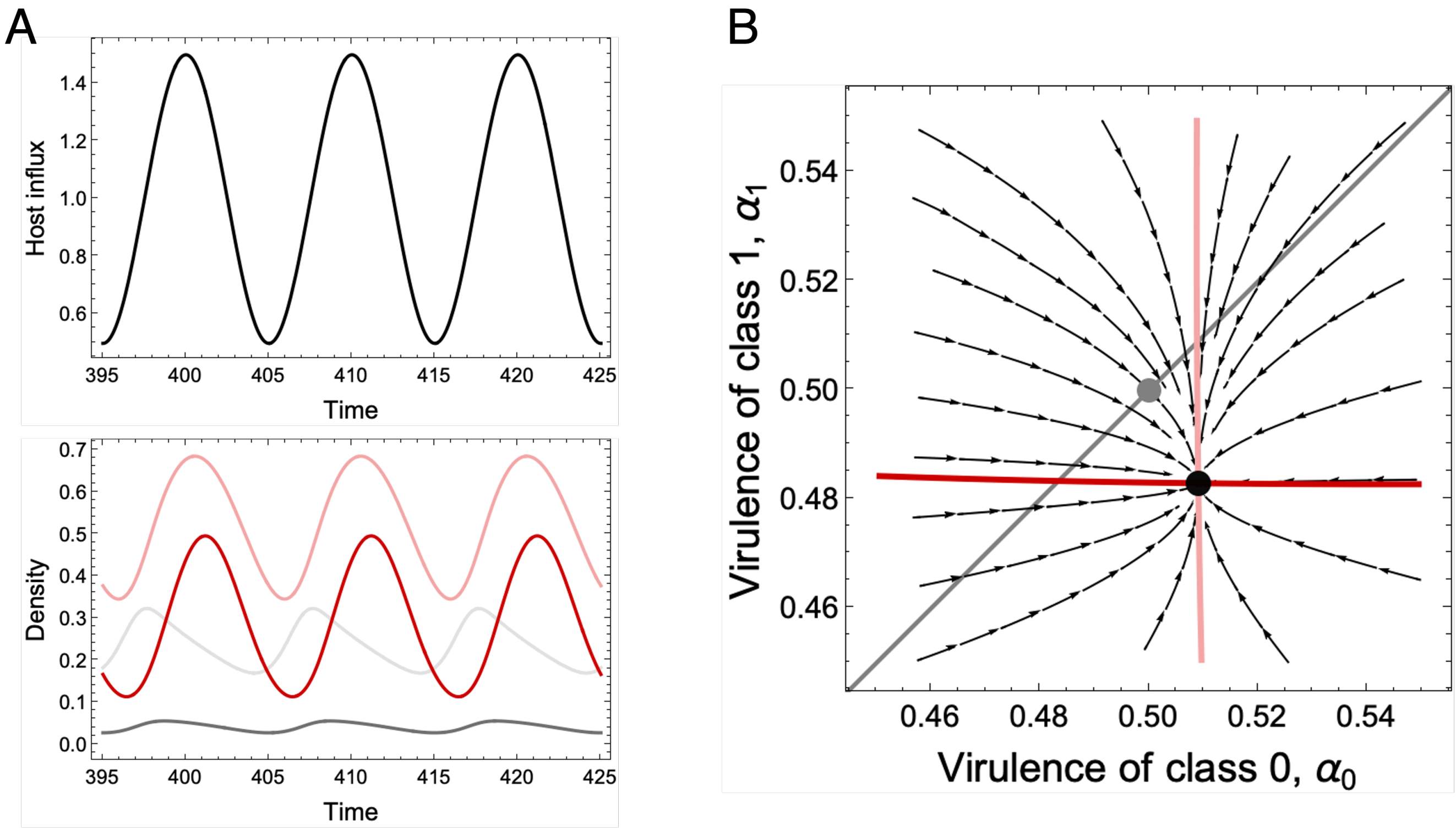

Fig. S3. Plastic virulence evolves under different temporal fluctuation. (A) Epidemiological dynamics are fluctuating (bottom) along with the host influx function $\theta(t) = \theta_0 + \delta\cos(2\pi t/T)$ (upper). (B) Selection towards plastic virulence under this temporal fluctuation. Colour code is the same as in Fig. 2. Other parameter values: $\theta_0 = 1, \beta_{max} = 5, \omega = 1, \gamma = 1, r = 1, d = 0.5, \delta = 0.5$.

Approximation for the covariance between information and susceptible hosts: The temporal covariance between $f_i(t)$ and $S(t)$, is key for the evolution of phenotypic plasticity. In the following, we use the perturbation analysis for the small period, which is used by (18), to obtain an analytic approximation of this covariance and to better understand the effect of various parameters on this covariance. In the following, we assume that the amplitude of the fluctuation is small ($\delta \ll 1$). In this case the epidemiological dynamics is fluctuating around the equilibrium $\{\hat{S}_0, \hat{S}_1, \hat{I}_0, \hat{I}_1\}$ reached without temporal fluctuations (when $\delta = 0$) in Eq. (25). A perturbation of the periodic attractors around this equilibrium point yields

$$S_0(t) = \hat{S}_0 + \delta\left[A_{S_0}\sin(\nu t) + B_{S_0}\cos(\nu t)\right] + o(\delta^2) \quad [57]$$

$$S_1(t) = \hat{S}_1 + \delta\left[A_{S_1}\sin(\nu t) + B_{S_1}\cos(\nu t)\right] + o(\delta^2) \quad [58]$$

$$I_0(t) = \hat{I}_0 + \delta\left[A_{I_0}\sin(\nu t) + B_{I_0}\cos(\nu t)\right] + o(\delta^2) \quad [59]$$

$$I_1(t) = \hat{I}_1 + \delta\left[A_{I_1}\sin(\nu t) + B_{I_1}\cos(\nu t)\right] + o(\delta^2) \quad [60]$$

We can determine the coefficients A_X and B_X by substituting these expressions into the epidemiological dynamics and retain only the first-order terms of δ. Since we need the covariance between $f_1(t)$ and $S(t)$, we can also write:

$$S(t) = \hat{S} + \delta\left(A_S\sin(\nu t) + B_S\cos(\nu t)\right) + o(\delta^2) \quad [61]$$

where $\hat{S} = \hat{S}_0 + \hat{S}_1$, $A_S = A_{S_0} + A_{S_1}$ and $B_S = B_{S_0} + B_{S_1}$ and:

$$A_S = \frac{\theta_0\nu^2\hat{S}}{\left((d+\alpha)^2\hat{I} - \hat{S}\nu^2\right)^2 + \nu^2\theta_0^2} \quad [62]$$

$$B_S = \frac{\hat{S}\nu(\hat{S}\nu^2 - \hat{I}(d+\alpha)^2)}{\left((d+\alpha)^2\hat{I} - \hat{S}\nu^2\right)^2 + \nu^2\theta_0^2} \quad [63]$$

After plugging equations (59) and (60) into the definition of frequency $f_1(t) = I_1(t)/(I_0(t) + I_1(t))$ and rearranging, we obtain

$$f_1(t) = \hat{f}_1 + \delta\left(A_F\sin(2\pi t/T) + B_F\cos(2\pi t/T)\right) + o(\delta^2) \quad [64]$$

where

$$A_F = \frac{A_{I_1}(1-\hat{f}_1) - A_{I_0}\hat{f}_1}{\hat{I}}, B_F = \frac{B_{I_1}(1-\hat{f}_1) - B_{I_0}\hat{f}_1}{\hat{I}} \quad [65]$$

Using the first order terms of equations (61) and (64), we obtain the following an approximation for the covariance:

$$\text{Cov}^{\text{T}}(f_1, S) \approx \frac{1}{T}\int S(t)f_1(t)dt - \hat{S}\hat{f}_1 \quad [66]$$

$$= \frac{\delta^2}{2}(A_S A_F + B_S B_F) := C_\delta \quad [67]$$

To discriminate the approximation from $\text{Cov}^{\text{T}}(f_1, S)$, we refer to the approximation as C_δ. The expression for this approximation is too long to be written down here but it will be available in a Mathematica notebook from a public depository before publication.

Covariance depends non-monotonically on environmental fluctuation period: The analytical approximation confirms that the covariance approaches zero in both the fast and slow fluctuation limits (see Mathematica notebook):

$$\lim_{T\to\infty} C_\delta = 0, \qquad \lim_{T\to 0} C_\delta = 0 \quad [68]$$

Consistent with numerical results under square-wave forcing (main text, Fig 2D), simulations with continuous forcing also show a unimodal relationship between covariance and the period T of environmental fluctuation (Fig. S4)

This pattern arises from the interplay between the timescales of environmental fluctuations and epidemiological dynamics. When $T \to 0$, environmental changes are too fast to affect infection dynamics and the dynamics always proceed as if the environment is in the averaged steady state even though it changes fast. On the other hand, when $T \to \infty$, the environment changes very slowly, allowing the system to reach rapidly a quasi-equilibrium. Because, the equilibrium density of susceptible hosts $\hat{S}$ is independent of θ (see Eq. (25)), so that there is no correlation between information and the density of susceptible hosts, and the covariance vanishes. Hence, the covariance vanishes when the environment fluctuates very fast or very slowly, but it peaks when these timescales are comparable, generating informative signals and stronger selection for plasticity.

Information dynamics also shapes covariance magnitude: We further examined the effect of signal-processing speed by rescaling signal production and decay rates:

$$\omega \to y\omega, \qquad \gamma \to y\gamma \quad [69]$$

where y controls the speed of information dynamics. We found that covariance is maximised at an intermediate value of y, with lower covariance observed when information dynamics are too fast ($y = 10$) or too slow ($y = 0.1$) (Fig. S4). We find that the covariance is maximized for an intermediate value of y and for lower values of β (Fig. S4). This indicates that the covariance, the factor that drives the evolution of plasticity in our model, is governed by (i) parameters governing the speed of the pathogen spread (i.e. β), (ii) parameters governing the speed of information spread (i.e. ω and γ) and (iii) the timescale of the environmental fluctuations (i.e. T).

Natural mortality affects the magnitude of covariance: We also investigated the effect of natural mortality rate d on the covariance $\text{Cov}^{\text{T}}(S, f_1)$ (Fig. S5). We found that the covariance is maximised at an intermediate value of d. The parameter d affects both the mean susceptible density $\hat{S}$ and the mean infected hosts $\hat{I}$ (Eq. (25)). When d is small, the average level of S is low, limiting the amplitude of fluctuations in $S(t)$ and thereby reducing the covariance. In contrast, when d is large, $\hat{S}$ increases but the infected density $\hat{I}$ decreases, making information transmission rare. As a result, the informed class becomes rare, which again reduces the covariance. Therefore, the covariance is expected to be maximized at an intermediate d, where there are enough susceptibles to generate variation in $S(t)$ and enough infected hosts to sustain information transmission.

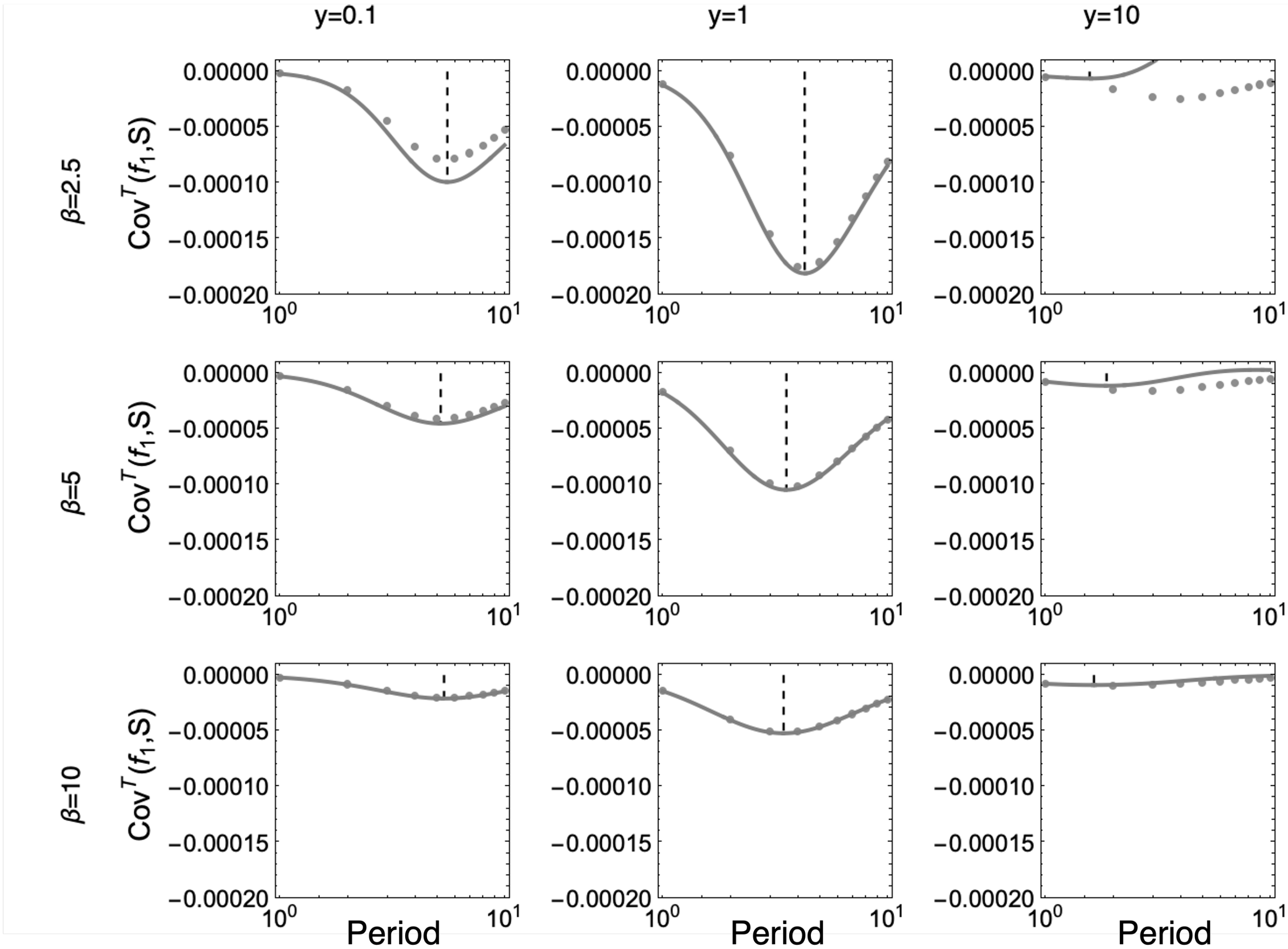

Fig. S4. The speed of the epidemiological and information processes modulate the peak and magnitude of the covariance between information and susceptible hosts. The covariance $\text{Cov}^T(S, f_1)$ (point) and its approximation C_δ (line) is plotted for different period of the environmental fluctuation. Transmission rate of the pathogens are varied as $\beta = 2.5$ (top), 5 (middle) and 10 (bottom) and the speed of information dynamics y is varied as $y = 0.1$ (left), 1 (center) and 10 (right). Black dashed line indicates the period which maximises C_δ. Other parameter values: $\theta_0 = 1, \beta_{max} = 5, \omega = 1, \gamma = 1, r = 1, d = 0.5, \delta = 0.1$.

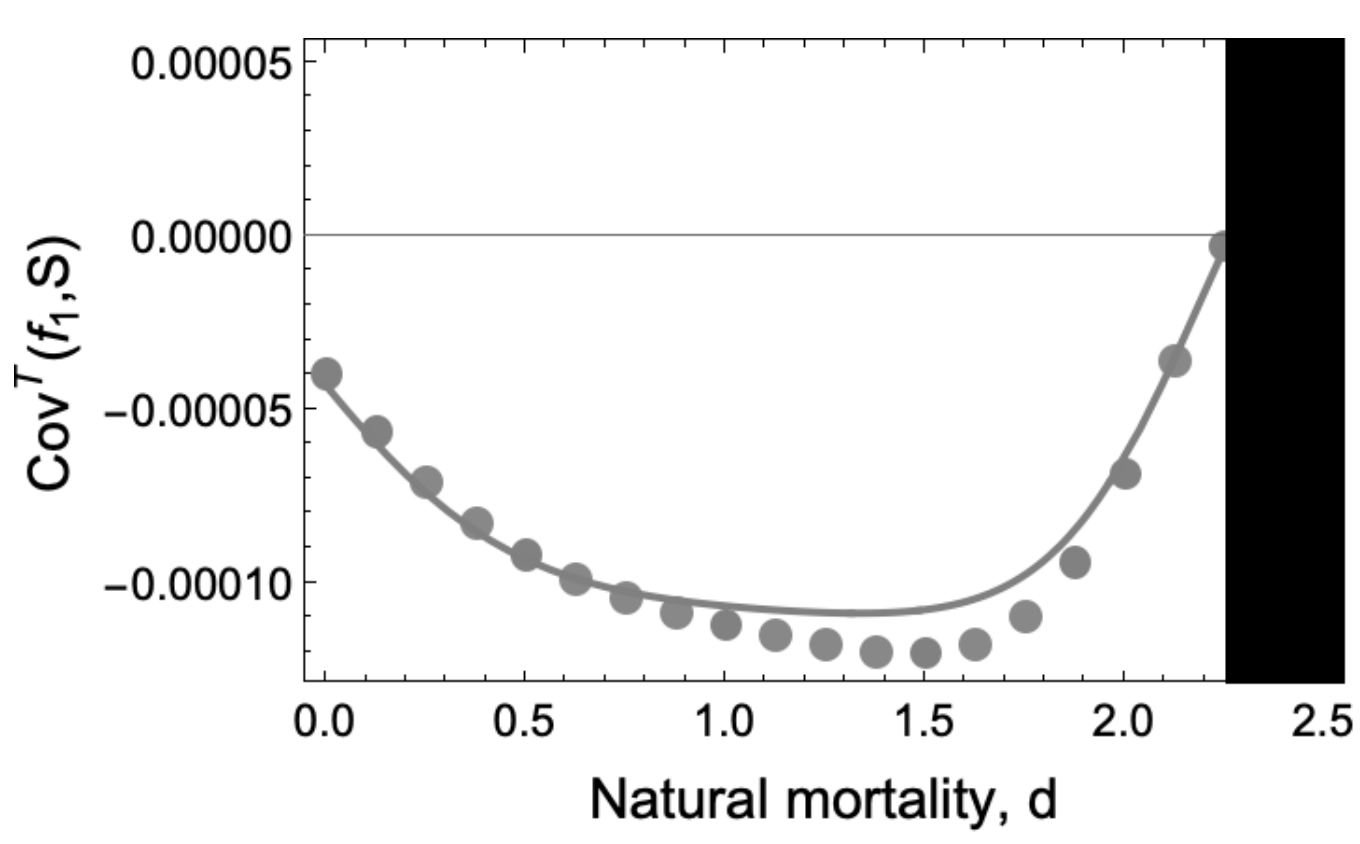

Fig. S5. Effect of the rate of host mortality on the magnitude of the covariance. The covariance $\text{Cov}^T(S, f_1)$ (point) and its approximation C_δ (line) is plotted for different values of the rate of host mortality d. Black area indicates the pathogens cannot be persist. Other parameter values: $\theta_0 = 1, \beta_{max} = 5, \omega = 1, \gamma = 1, r = 1, d = 0.5, \delta = 0.1$.

4. Spatially structured model

In this section we analyse how spatial structure affects the selection for plasticity. We assume that the epidemiological dynamics takes place on a regular square lattice where each site can be either empty (denoted by $\emptyset$) or occupied by one host with state

S_0, S_1, I_0 or I_1 (Fig. 1). As explained in the main text, the parameter D (for dispersal) governs the degree of spatial structure: global events occur with probability D, and local events occur with probability of $1-D$. Global events depend on the average density of different types of sites. Local events, in contrast, depend on the state of neighboring sites. In particular, three key events (host reproduction, information transmission, and pathogen transmission) are influenced by the spatial structure.

A. Spatial epidemiological dynamics with information. We note p_X the density of sites in state $X \in \{\emptyset, S_0, S_1, I_0, I_1\}$ which yields the following dynamical equations

$$\begin{aligned}
\frac{dp_{S_0}}{dt} &= b[\emptyset|S]p_S + b[\emptyset|S_1]p_{S_1} - \beta_0[S_0|I_0]p_{I_0} - \beta_1[S_0|I_1]p_{I_1} - \omega[I|S_0]p_{S_0} - dp_{S_0} + \gamma p_{S_1} \\
\frac{dp_{S_1}}{dt} &= -\beta_0[S_1|I_0]p_{I_0} - \beta_1[S_1|I_1]p_{I_1} + \omega[I|S_0]p_{S_0} - dp_{S_1} - \gamma p_{S_1} \\
\frac{dp_{I_0}}{dt} &= \beta_0[S_0|I_0]p_{I_0} + \beta_1[S_0|I_1]p_{I_1} - \omega[I|I_0]p_{I_0} - (d+\alpha_0)p_{I_0} + \gamma p_{I_1} \\
\frac{dp_{I_1}}{dt} &= \beta_0[S_1|I_0]p_{I_0} + \beta_1[S_1|I_1]p_{I_1} + \omega[I|I_0]p_{I_0} - (d+\alpha_1)p_{I_I} - \gamma p_{I_1}
\end{aligned}$$

with the effective density of site X for site Y

$$[X|Y] = (1-D)\, q_{X/Y} + D\, p_X \tag{70}$$

where $q_{X/Y}$ is the conditional probability that site X appears in the neighborhood of site Y. Because $p_\emptyset$ is determined by the constraint that the total number of sites is constant $\sum p_X = 1$, its dynamics is obtained from the sum of the above four dynamical equations.

B. Evolution of virulence in a spatially structured model. In the spatially structured population, the mutant invasion dynamics is described by spatial moment equations (5, 19, 20). To see this, we first consider the invasion of the mutant without information in the spatially structured population. In the absence of information (i.e. $\omega = 0$) the invasion dynamics of the mutant I^m with phenotypic values $(\beta(\alpha^m), \alpha^m)$ into the ecological equilibrium of the resident is (21–23)

$$\frac{dp_{I^m}}{dt} = \beta(\alpha^m)[S|I^m]p_{I^m} - (d+\alpha^m)p_{I^m} = \lambda^m p_{I^m} \tag{71}$$

where $[S|I^m] = (1-D)q_{S/I^m} + D\, p_S$ is the effective susceptible density for the mutant and λ^m is the growth rate of mutant. In the spatially structured population, the local density around a rare mutant has a fast dynamics and rapidly converges to a quasi-steady state (19, 24). Then, we assume q_{S/I^m} is at the quasi-equilibrium as well as p_S is at the equilibrium of the resident dynamics, which yields the linearized dynamics of the mutant invasion. Note that the above dynamics (Eq. (71)) is the expected dynamics of the fraction of sites infected by the mutant in the spatially explicit population (19).

The long-term success of the mutant depends on the sign of the invasion fitness λ^m: the mutant can invade only if λ^m is positive. Assuming weak selection, we can derive the selection gradient, which is given by the derivative of the invasion growth rate by the mutant trait $\partial\lambda^m/\partial\alpha^m$ (11). Then we recover the selection gradient obtained in previous studies of virulence evolution in spatially structured population (21, 22):

$$\mathcal{S}^{\mathrm{S}} = \underbrace{\beta'(\alpha)[S|I] - 1}_{\text{trade-off}} + \underbrace{(1-D)\beta\frac{\partial q_{S/I^m}}{\partial \alpha^m}}_{\substack{\text{local susceptible density} \\ \text{around mutant}}} \tag{72}$$

where the second term depends on $\frac{\partial q_{S/I^m}}{\partial \alpha^m}$ which quantifies the effect of the mutation on the local density of susceptible hosts in the neighbourhood of a host infected by the mutant. This term is usually expected to be negative because, increasing virulence reduces the density of hosts.

C. Evolution of class specific virulence in spatially structured population. The dynamics of the rare mutants with class-specific virulence (α_0^m, α_1^m) is given by the following dynamical equations

$$\frac{dp_{I_0^m}}{dt} = \beta_0^m[S_0|I_0^m]p_{I_0^m} + \beta_1^m[S_0|I_1^m]p_{I_1^m} - \omega([I|I_0^m] + [I^m|I_0^m])p_{I_0^m} - (d+\alpha_0^m)p_{I_0^m} + \gamma p_{I_1^m} \tag{73}$$

$$\frac{dp_{I_1^m}}{dt} = \beta_0^m[S_1|I_0^m]p_{I_0^m} + \beta_1^m[S_1|I_1^m]p_{I_1^m} + \omega([I|I_0^m] + [I^m|I_0^m])p_{I_0^m} - (d+\alpha_1^m)p_{I_1^m} - \gamma p_{I_1^m} \tag{74}$$

and the mutant invasion matrix $\mathbf{M}^m$ is

$$\mathbf{M}^m = \begin{pmatrix} \beta(\alpha_0^m)[S_0|I_0^m] - (d+\alpha_0^m) - \omega([I|I_0^m] + [I^m|I_0^m]) & \beta(\alpha_1^m)[S_0|I_1^m] + \gamma \\ \beta(\alpha_0^m)[S_1|I_0^m] + \omega([I|I_0^m] + [I^m|I_0^m]) & \beta(\alpha_1^m)[S_1|I_1^m] - (d+\alpha_1^m+\gamma) \end{pmatrix} \tag{75}$$

In the same way, the dynamics of the resident with class-specific virulence (α_0, α_1) is represented by the linearised matrix

$$\mathbf{M} = \begin{pmatrix} \beta(\alpha_0)[S_0|I_0] - (d+\alpha_0) - \omega([I|I_0]) & \beta(\alpha_1)[S_0|I_1] + \gamma \\ \beta(\alpha_0)[S_1|I_0] + \omega([I|I_0] + [I|I_0]) & \beta(\alpha_1)[S_1|I_1] - (d+\alpha_1+\gamma) \end{pmatrix} \quad [76]$$

Again, recall the local density around a rare mutant have a fast dynamics that rapidly converges to a quasi-equilibrium (19, 24). Assuming the $q_{X/Y}$ have settled onto this quasi-equilibium value, the mutant dynamics become linear and invasion is determined by the leading eigenvalue of the linearised matrix $\mathbf{M}^m$. Note that we evaluate $\mathbf{M}^m$ at the quasi-equilibrium that is reached in the mutant invasion.

Frequency-based selection gradient: Following the definition in Eq. (13), the spatial selection gradient for class-specific virulence is

$$\mathcal{S}_i^{\mathrm{S}} = f_i\left(\beta'(\alpha_i)[S|I_i] - 1\right) + \Delta\rho\frac{\partial f_1^m}{\partial \alpha_i^m} + (1-D)\sum_{j=0,1}\beta_j f_j \frac{\partial q_{S/I_j^m}}{\partial \alpha_i^m} \quad [77]$$

In the first term of the above gradient, the covariance between information and accessible susceptible host is hidden. The first term can be arranged as

$$f_i\left(\beta'(\alpha_i)[S|I_i] - 1\right) = f_i\left(\beta'(\alpha_i)[S|I] - 1\right) + (1-D)\beta'(\alpha_i) f_i\left(q_{S/I_i} - q_{S/I}\right)$$

$f_i\left(q_{S/I_i} - q_{S/I}\right)$ in the second term in the RHS of the above equation can be rearranged as

$$f_i\left(q_{S/I_i} - q_{S/I}\right) = \frac{p_{SI_i}}{p_I} - \frac{p_{SI}}{p_I}\frac{p_{I_i}}{p_I} \quad [78]$$

We introduce the index variable for the host state, $\sigma^X(x)$ defined as

$$\sigma^X(x) = \begin{cases} 1 & x \text{ is in state } X \in [S, I] \\ 0 & \text{otherwise} \end{cases} \quad [79]$$

and the index variable for the information class, $\sigma_i(x)$ defined as

$$\sigma_i(x) = \begin{cases} 1 & x \text{ is in class } i \in [0, 1] \\ 0 & \text{otherwise} \end{cases} \quad [80]$$

We note $n_S(x)$ the proportion of S sites among the 4 neighboring sites of the focal site x which yields the following relationships:

$$\begin{aligned} \frac{p_{SI_i}}{p_I} &= \frac{\sum_x n_S(x)\sigma_i(x)\sigma^I(x)}{\sum_x \sigma^I(x)} = \sum_x n_S(x)\sigma_i(x)\pi(x) = E_\pi(n_S\,\sigma_i) \\ \frac{p_{SI}}{p_I} &= \frac{\sum_x n_S(x)\sigma^I(x)}{\sum_x \sigma^I(x)} = \sum_x n_S(x)\pi(x) = E_\pi(n_S) \\ f_i = \frac{p_{I_i}}{p_I} &= \frac{\sum_x \sigma_i(x)\sigma^I(x)}{\sum_x \sigma^I(x)} = \sum_x \sigma_i(x)\pi(x) = E_\pi(\sigma_i) \end{aligned} \quad [81]$$

where $\pi(x) = \sigma^I(x)/\sum \sigma^I(x)$ is the spatial distribution of infected hosts and E_π means taking the average over π. Plugging the above relationships into Eq. (78), the covariance appears as

$$f_i\left(q_{S/I_i} - q_{S/I}\right) = E_\pi(\sigma_i\,n_S) - E_\pi(\sigma_i)E_\pi(n_S) = \mathrm{Cov}^{\mathrm{S}}(\sigma_i, n_S) \quad [82]$$

This measures the covariance in space between the information and available susceptible hosts around infected hosts. This finally yields the following expression for selection gradient in a spatially structured environment

$$\mathcal{S}_i^{\mathrm{S}} = \underbrace{f_i\left(\beta'(\alpha_i)[S|I] - 1\right)}_{\mathcal{T}_i^{\mathrm{S}}} + \underbrace{\Delta\rho\frac{\partial f_1^m}{\partial \alpha_i^m}}_{\mathcal{F}_i^{\mathrm{T}}} + \underbrace{(1-D)\sum_{j=0,1}\beta_j f_j \frac{\partial q_{S/I_j^m}}{\partial \alpha_i^m}}_{\mathcal{Q}_i^{\mathrm{S}}} + \underbrace{(1-D)\beta'(\alpha_i)\mathrm{Cov}^{\mathrm{S}}(\sigma_i, n_S)}_{\mathcal{C}_i^{\mathrm{S}}} \quad [83]$$

RV-based selection gradient: Using Eq. (14), we can derive an equivalent but RV-based spatial selection gradient for class-specific virulence

$$\begin{aligned}\mathcal{S}_i^{\mathrm{S}} =& f_i\left(\beta'(\alpha_i)[S_v|I]-1\right)+(1-D)\omega f_0(v_1-v_0)\left(\frac{\partial q_{I/I_0^m}}{\partial \alpha_i^m}+\frac{\partial q_{I^m/I_0^m}}{\partial \alpha_i^m}\right)\\ &+(1-D)\sum_{j=0,1}\beta_j f_j\frac{\partial q_{S_v/I_j^m}}{\partial \alpha_i^m}+(1-D)\beta'(\alpha_i)\mathrm{Cov}^{\mathrm{S}}(\sigma_i,n_{S_v})\end{aligned} \quad [84]$$

where $S_v = v_0S_0 + v_1S_1$, $q_{S_v/I_j} = v_0q_{S_0/I_j} + v_1q_{S_1/I_j}$. The covariance term is defined as

$$\mathrm{Cov}^{\mathrm{S}}(\sigma_i, n_{S_v}) = E_\pi(\sigma_i\, n_{S_v}) - E_\pi(\sigma_i)E_\pi(n_{S_v}) \quad [85]$$

with $n_{S_v} = v_0n_{S_0} + v_1n_{S_1}$ and where n_{S_i} is the proportion of S_i hosts among the 4 neighbouring sites of the focal site.

D. Pair approximation. Pair approximation is a type of moment closure that closes a hierarchical system of moment equations by approximating higher-order moments (three-site correlation) with two-site correlation (pair correlation) (19, 20, 23–26). We use this pair approximation to simplify the numerical evaluation of the spatial selection gradient (Eq. (83)). Let p_X be the density of the sites in state X and p_{XY} the density of pairs XY. Each site is in one of the seven states $\{\emptyset, S_0, S_1, I_0, I_1, I_0^m, I_1^m\}$. We track the dynamics of pair densities instead of the site density. The dynamics of pairs depends on the conditional probability, $q_{X/YZ}$, which is the probability that site X is in the neighborhood of the Y site in a YZ pair. To know $q_{X/YZ}$, one needs to know the density of triplet p_{XYZ} as $q_{X/YZ} = p_{XYZ}/p_{YZ}$. In other words, the description of the dynamics of pair densities depends on the state of neighbors located more than one step away. The pair approximation neglects the influence of more distant neighbors and replaces $q_{X/YZ}$ by $q_{X/Y}$ as

$$q_{X/YZ} \approx q_{X/Y} \quad [86]$$

E. Covariance in space or in time. In the temporally variable environment we show that what drives the evolution of plasticity is the temporal covariance between $f_i(t)$ and $S(t)$

$$\begin{aligned}\mathrm{Cov}^{\mathrm{T}}(f_i, S) &= \frac{1}{T}\int_{t_a}^{t_a+T} f_iS dt - \left(\frac{1}{T}\int_{t_a}^{t_a+T} f_i dt\right)\left(\frac{1}{T}\int_{t_a}^{t_a+T} S dt\right)\\ &= E_T(f_iS) - E_T(f_i)E_T(S)\end{aligned}$$

where the integral is calculated over on period of the fluctuation between t_a and $t_a + T$, where t_a is chosen so that the system is sitting on its periodic attractor. The notation, $E_T(X)$ refers to the temporal average of the dynamical variable $X(t)$.

In the spatial model, there is a duality between the spatial covariance for the entire grid and the temporal covariance for each site. We have ensembles of the spatial dynamics on the spatial domain χ and the temporal domain $\tau = [t_1, t_2]$. Here, we denote the index variable $\sigma^I(x, t)$ with explicit space and time dependency. The spatial distribution of infected hosts at a given time t is defined as

$$\pi_S(x, t) = \frac{\sigma^I(x, t)}{\sum_{x\in\chi}\sigma^I(x, t)}$$

and the spatial covariance between $\sigma_i(x, t)$ and $n_S(x, t)$ at a given time t

$$\begin{aligned}\mathrm{Cov}_t^{\mathrm{S}}(\sigma_i, n_S) &= \frac{\sum_{x\in\chi} n_S(x,t)\sigma_i(x,t)\sigma^I(x,t)}{\sum_{x\in\chi}\sigma^I(x,t)} - \frac{\sum_{x\in\chi} n_S(x,t)\sigma^I(x,t)}{\sum_{x\in\chi}\sigma^I(x,t)}\frac{\sum_{x\in\chi}\sigma_i(x)\sigma^I(x,t)}{\sum_{x\in\chi}\sigma^I(x,t)}\\ &= E_\pi^{\mathrm{S}}(\sigma_i\, n_S) - E_\pi^{\mathrm{S}}(\sigma_i)E_\pi(n_S)\end{aligned}$$

and its expectation over time

$$\mathrm{Cov}^{\mathrm{S}}(\sigma_i, n_S) = \frac{1}{|\tau|}\sum_{t\in\tau}\mathrm{Cov}_t^{\mathrm{S}}(\sigma_i, n_S).$$

This is consistent with the spatial covariance in the selection gradient (Eq. (83)).

Interestingly, we can also compute a temporal covariance in the spatially structured environment. Let $\pi_T(x, t)$ be the distribution of infected hosts at site x over the temporal domain $\tau = [t_1, t_2]$ as defined by

$$\pi_T(x, t) = \frac{\sigma^I(x, t)}{\sum_{t\in\tau}\sigma^I(x, t)}$$

If we focus on a given site x we can compute the following temporal covariance

$$\mathrm{Cov}_x^{\mathrm{T}}(\sigma_i, n_S) = \frac{\sum_{t_1}^{t_2} n_S(x)\sigma_i(x)\sigma^I(x)}{\sum_{t_1}^{t_2}\sigma^I(x)} - \frac{\sum_{t_1}^{t_2} n_S(x)\sigma^I(x)}{\sum_{t_1}^{t_2}\sigma^I(x)}\frac{\sum_{t_1}^{t_2}\sigma_i(x)\sigma^I(x)}{\sum_{t_1}^{t_2}\sigma^I(x)}$$

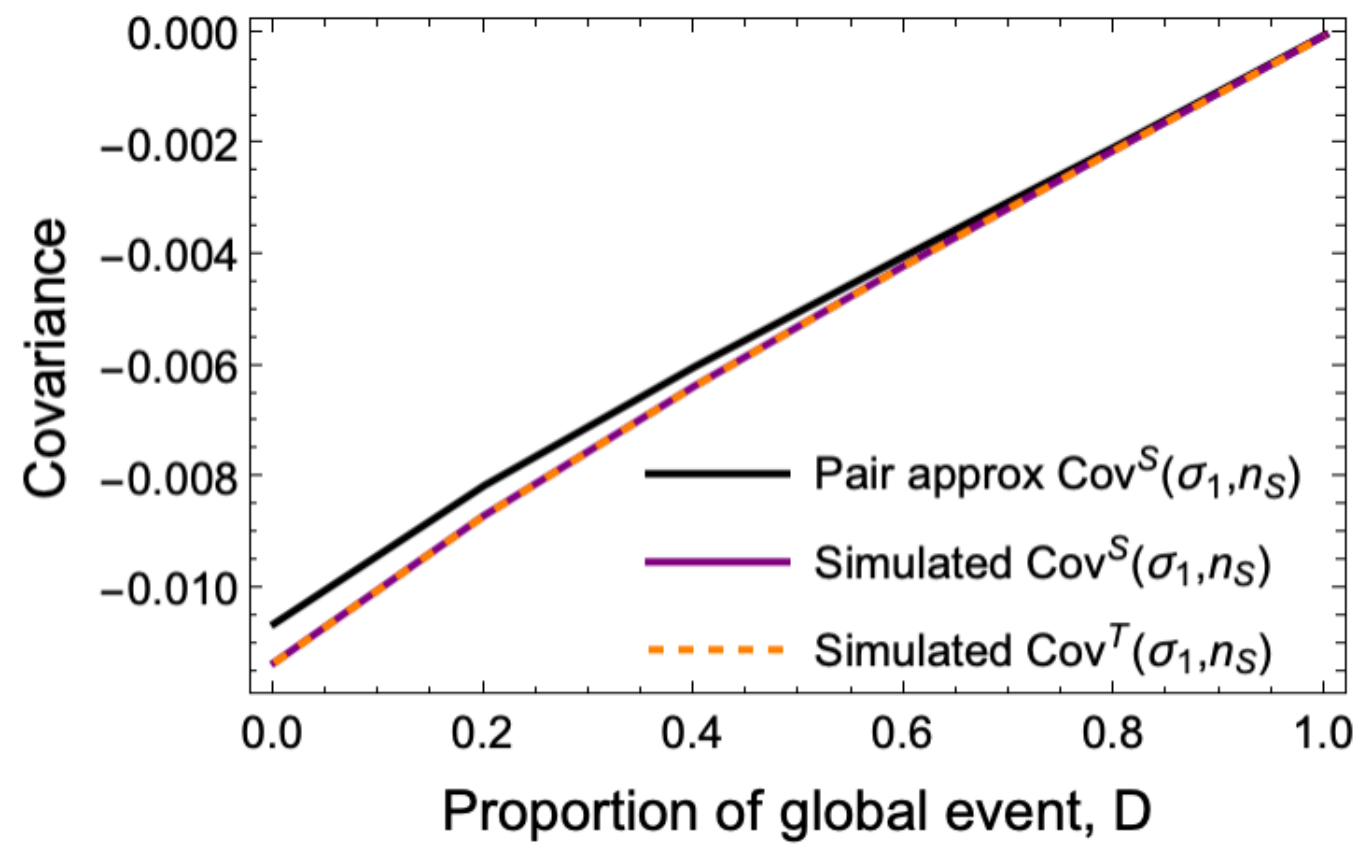

Fig. S6. Temporal covariance generated in the spatially structured population greatly matches the spatial covariance. Black line is given by the spatial covariance expected by the pair approximation. Purple line is the average value of spatial covariance $\mathrm{Cov}^{\mathrm{S}}(\sigma_i, n_S)$ over the timepoints from $t = 5000$ to $t = 10000$. Orange dashed line is the temporal covariance $\mathrm{Cov}_x^{\mathrm{S}}(\sigma_i, n_S)$ calculated from the ensembles from $t = 5000$ to $t = 10000$ for each site and then averaged over space.

$$= E_\pi^{\mathrm{T}}(\sigma_i\, n_S) - E_\pi^{\mathrm{T}}(\sigma_i) E_\pi^{\mathrm{T}}(n_S)$$

and its expectation over space

$$\mathrm{Cov}^{\mathrm{T}}(\sigma_i, n_S) = \frac{1}{|\chi|} \sum_{x \in \chi} \mathrm{Cov}_x^{\mathrm{T}}(\sigma_i, n_S).$$

We numerically find that averaging over space and then over time yields the same result as averaging over time and then over space so that $\mathrm{Cov}^{\mathrm{S}}(\sigma_i, n_S) = \mathrm{Cov}^{\mathrm{T}}(\sigma_i, n_S)$ (Fig. S6). This equivalence between the temporal covariance and the spatial covariance stems from the fact that our spatial model has ergodicity.

Note that for the pair approximation model we can only compute the spatial covariance because when we model the dynamics of the densities of pairs of sites, these densities have already been averaged over space. The covariance obtained in the stochastic model yield very similar results as the covariance obtained in the pair approximation model (Fig. S6). Pair approximation considers the correlation between two adjacent individuals, but ignores higher-order spatial structures such as loops. On the other hand, lattice models in the simulations naturally reflect higher-order spatial structures such as self-looping, local interactions and cluster formation. The quantitative differences in Fig. 3E reflects this difference between the two approaches. This difference is also reflected in the evolutionary results described in the main text.

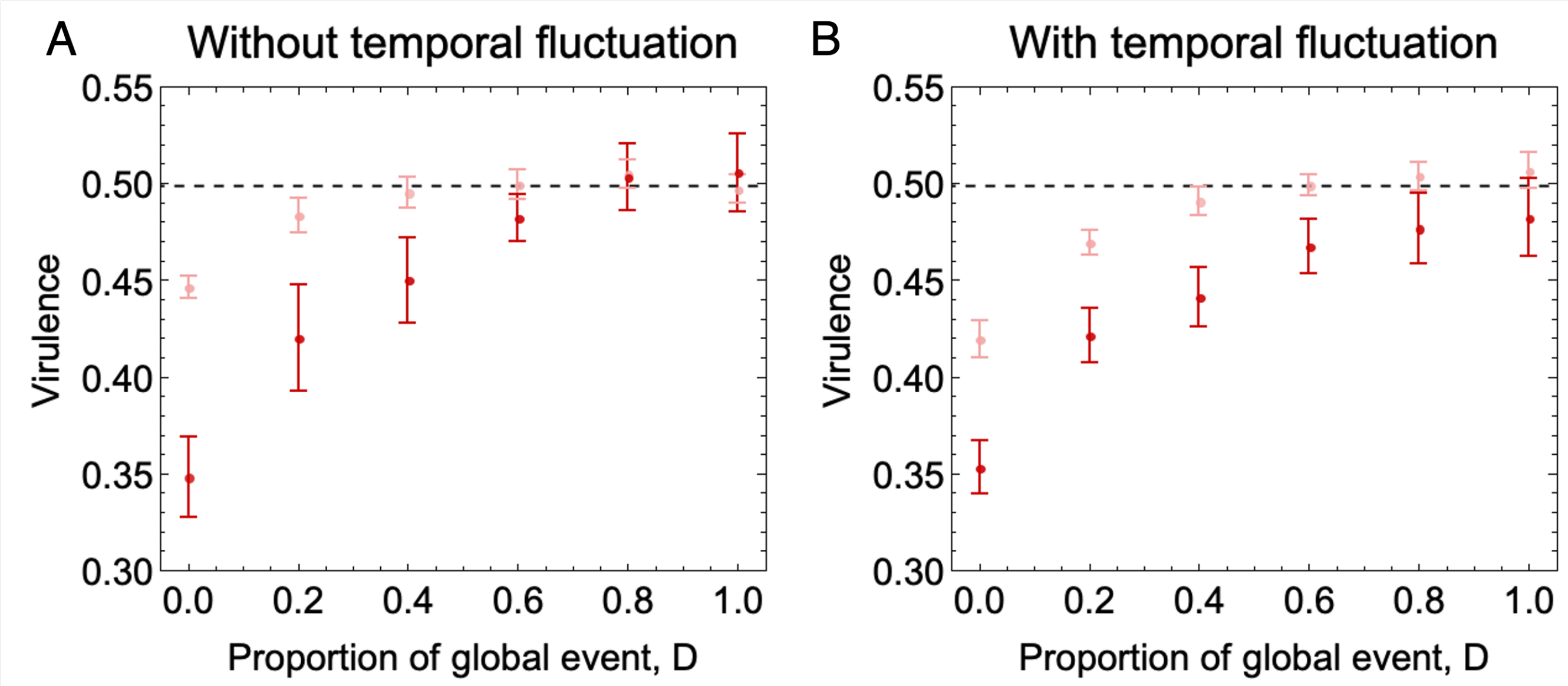

Fig. S7. Interaction between the effect of spatial and temporal variation on the evolution pathogen plasticity. The results of evolved virulence in spatial IBM simulations without (A) and with (B) a temporally fluctuating host influx $\theta(t)$. In B, we model the temporal host influx as the rate of host influx switches between $\theta_A = 1$ and $\theta_B = 10$ in every time 5 passes (Period $T = 10$) as the same in the temporally fluctuating scenario used in the main text (Fig. 2). Average virulence ($\pm$ SD) obtained from 10 independent simulations for different values of dispersal D. Panel A is the same in Fig. 3E. Parameter values: $r = 1, \theta_A = 10, \theta_B = 1, T = \beta_{max} = 5, d = 0.5, \omega = 1, \gamma = 1$.

F. Individual based simulations. We carried out stochastic individual-based simulations for the spatially structured model using a square grid of $100 \times 100 = 10,000$ sites (unless otherwise specified). We used a Gillespie algorithm (27) to model the different types of events that may take place in the population (e.g. reproduction, death, transmission of the infection, transmission of the information...). The phenotype of each infected individual is characterised by a pair of virulence traits (α_0 and α_1) which also affects transmission rates via the virulence-transmission function $\beta(\alpha)$. Crucially, we allow each virulence trait to mutate at a constant rate $\mu = 0.05$ and the new trait value is increased or decreased by 0.01 (randomly). Fig. 3E, S7, and S8 show the average values ($\pm$ SD) of the virulence traits over 5000 time steps (after a burn-in period of 5000 time steps) of 10 independent simulations. The confidence intervals are computed from this 10 independent replicates. The simulation code will be available on a public repository upon publication.

G. Effect of temporal fluctuations of host influx. In the main text, we assume that the external host influx is absent in the spatial IBM model ($\theta(t) = 0$). We modified our individual-based simulation model to explore how an external influx of host could affect our results in a spatially structured population We numerically integrated the host influx from outside and we investigated the effect of temporal fluctuations in the spatial individual-based simulations (Fig. S7). In the presence of temporally fluctuating host influx, α_0 and α_1 evolve toward different value (i.e. plasticity is adaptive) even in the well-mixed population ($D = 1$). This is consistent with the result of temporally fluctuating population discussed in section S2 where the temporal covariance created by a periodic host influx drives the evolution of plasticity. In the spatially structured population ($D < 1$), adding a periodic influx of host has only a minor quantitative effect on the evolution of pathogen plasticity. When $D = 0$ the virulence α_0 evolves around 0.45 without temporal fluctuations (Fig. S7A), but it evolves to a lower value (0.42) with temporal fluctuation (Fig. S7B) and gets closer to α_1. This reduction of the amplitude of plasticity suggests that the combination of spatial and temporal fluctuations interfere with each other which reduces the value carried by the social cue. This interference, however, remains to be investigated analytically.

H. Effect of demographic stochasticity. In the individual-based model, population size is finite and equal to L, number of sites in the square lattice. This finite population size can generate demographic stochasticity and can affect pathogen evolution (28, 29). To check whether demographic stochasticity affects the evolution of plasticity, we carried out the individual-based simulations with different lattice sizes (Fig. S8). We found that using a smaller lattice size increases the phenotypic variance but there is no evidence of an effect on the evolution of plasticity when $D = 1$ (similar results were obtained for $D = 0$). These results suggest that the predictions we obtain from an analysis of the deterministic models are robust and that demographic stochasticity has a limited influence on the evolution of pathogen plasticity.

5. Alternative life-cycle: SIR dynamics

For simplicity, we focused on an SI model, but several extensions are possible. As an illustration, we consider an SIR model in which hosts recover and acquire perfect immunity (Fig. S9A), and we analyze the evolution of plasticity in a temporally fluctuating environment. Although this changes the host life cycle, the selection gradient for class-specific virulence is unchanged.

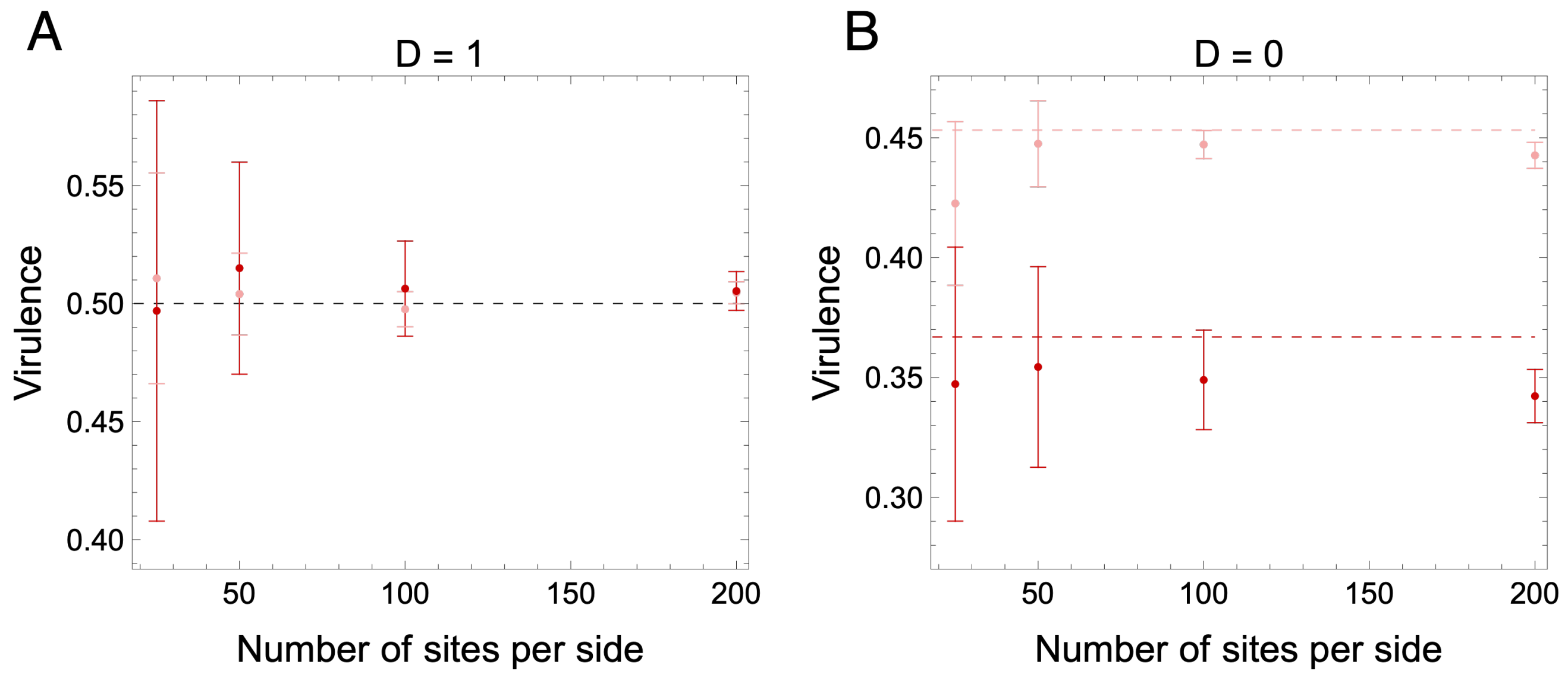

Fig. S8. The effect of lattice size on the evolution of plastic virulence. Spatial individual-based simulations are carried out changing the number of lattice size L ($L = 25 \times 25, 50 \times 50, 100 \times 100$, and 200×200). Average virulence ($\pm$ SD) obtained from 10 independent simulations for different values of dispersal D. Dashed lines indicate the prediction of ESS by the selection gradients from the pair approximation (section S4). Parameter values: $r = 10, \beta_{max} = 5, d = 0.5, \omega = 1, \gamma = 1$.

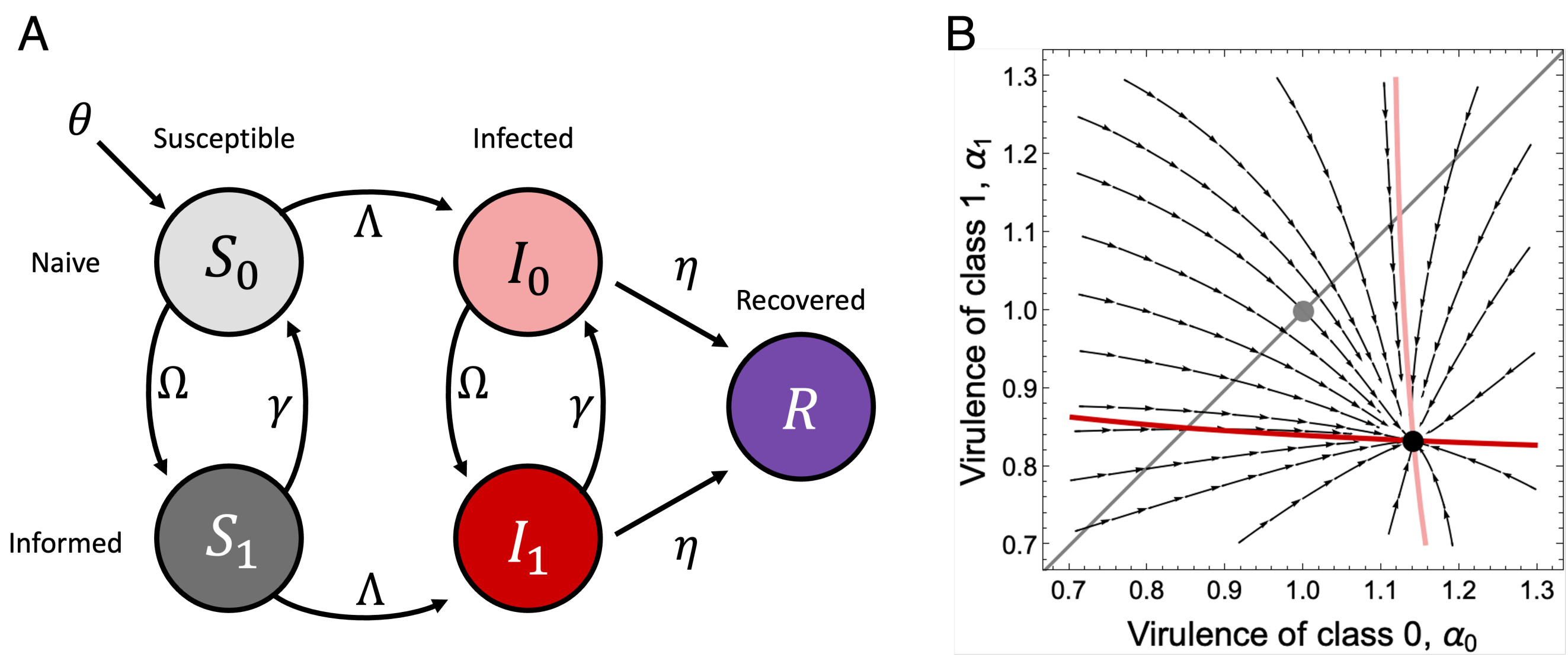

Fig. S9. Plastic virulence evolves in SIR model with temporally fluctuating host influx. A. Graphical diagram of the SIR model with information. B. Selection towards plastic virulence under a scenario with temporal fluctuations $\theta(t)$. Colour code is the same as in Fig. 2. Other parameter values: $\theta_0 = 1, \beta_{max} = 5, \omega = 1, \gamma = 1, r = 1, d = 0.5, \delta = 0.5, \eta = 1$.

Specifically, the mutant invasion matrix becomes

$$\mathbf{M}^m_{\text{SIR}}(t) = \begin{pmatrix} \beta(\alpha_0^m)S_0(t) - (d + \alpha_0^m + \eta) - \omega I(t) & \beta(\alpha_1^m)S_0(t) + \gamma \\ \beta(\alpha_0^m)S_1(t) + \omega I(t) & \beta(\alpha_1^m)S_1(t) - (d + \alpha_1^m + \eta + \gamma) \end{pmatrix} \quad [87]$$

$$= \mathbf{M}^m(t) - \eta \mathbf{I}, \quad [88]$$

where $\mathbf{M}^m(t)$ is the invasion matrix in the original model (Eq. (41)) and $\mathbf{I}$ is the identity matrix. Thus, recovery adds a trait-independent loss rate η to infected classes, shifting the invasion matrix by $-\eta\mathbf{I}$ without affecting its trait derivatives. Consequently, the class-specific selection gradient is identical to that of the SI model, and plasticity still evolves in temporally fluctuating populations (Fig. S9B).

6. Numerical recipes for the selection gradients

This section outlines the numerical procedures used to compute the selection gradients under two scenarios: temporally fluctuating environments and spatially structured populations. The selection gradient involves the evaluation of two key derivative terms: the derivative of the class frequency, $\frac{\partial f_i^m}{\partial \alpha_i^m}$, and the derivative of the local density of susceptible hosts around an infected individual, $\frac{\partial q_{S/I_i^m}}{\partial \alpha_i^m}$. The latter appears only in the spatial model. Both terms are calculated as deviations from the values obtained for neutral mutants—mutants with identical traits to the resident population.

Step1. Calculate the resident dynamics

We assume that the resident parasite with trait values (α_0, α_1) is at ecological equilibrium. To begin, we numerically simulate the resident dynamics for a sufficiently long time to reach this equilibrium. In the temporally fluctuating case, this corresponds to a periodic attractor; in the spatial case, it corresponds to a stationary distribution of pair densities.

Step2. Calculate the neutral mutant dynamics

Once equilibrium is reached, we introduce a neutral mutant by replacing 1% of the resident population with individuals that share the same trait values as the resident ($\alpha_0^m = \alpha_0$, $\alpha_1^m = \alpha_1$). Starting from this mixed population, we simulate the system again and evaluate the dynamics of the neutral mutant. From this, we calculate the class frequency of the neutral mutant, f_i. In the spatial model, we also compute the local density of susceptible hosts around the neutral mutant, q_{S/I_i}. These quantities are used as reference values for calculating selection gradients. If one uses the reproductive value–based formulation of the gradient (Eq. (50) and Eq. (84)), the reproductive values can also be derived from the linearized dynamics of the neutral mutant population as shown in (8).

Step3. Calculate the mutant dynamics

To evaluate the actual selection gradient, we introduce a mutant with a small deviation in one of the trait components. For instance, to calculate the gradient with respect to α_0, we simulate the invasion of a mutant with $(\alpha_0^m = \alpha_0 + \epsilon, \alpha_1^m = \alpha_1)$; for the gradient with respect to α_1, we use $(\alpha_0^m = \alpha_0, \alpha_1^m = \alpha_1 + \epsilon)$. Here, ϵ is a small perturbation (throughout this study, we use $\epsilon = 0.01$), reflecting the assumption of weak selection. As in step 2, we simulate the dynamics of the resident and the mutant starting from mixed equilibrium with 1% of resident equilibrium density replaced by the mutant. We compute the equilibrium class frequency f_i^m of the mutant, and in the spatial model, the local susceptible density around mutant individuals, q_{S/I_i^m}.

Step4. Evaluate the components of the selection gradients

We can evaluate the differentiation terms of f_i^m as

$$\frac{\partial f_i^m}{\partial \alpha_i^m} \approx \frac{f_i^m - f_i}{\alpha_i^m - \alpha_i} = \frac{f_i^m - f_i}{\epsilon}$$

and that of q_{S/I_i^m} as

$$\frac{\partial q_{S/I_i^m}}{\partial \alpha_i^m} \approx \frac{q_{S/I_i^m} - q_{S/I_i}}{\alpha_i^m - \alpha_i} = \frac{q_{S/I_i^m} - q_{S/I_i}}{\epsilon}$$

Finally, we can calculate the selection gradient in Eq. (48) and Eq. (83) with the above terms.

7. Full pair dynamics

Using spatial moment equation (19), we can write down the spatial dynamics of resident and mutant pathogens with a set of 28 pair dynamics:

$$\begin{aligned}
\frac{dp_{S_0S_0}}{dt} &= \left(\mathcal{H} + (1-D)\frac{b}{n}\right) p_{S_0\emptyset} - 2(\mathcal{B}_{S_0} + \mathcal{B}^m_{S_0})p_{S_0S_0} - 2\mathcal{I}_{S_0}p_{S_0S_0} - 2dp_{S_0S_0} + \gamma p_{S_0S_1}\\
\frac{dp_{S_0S_1}}{dt} &= \left(\mathcal{H} + (1-D)\frac{b}{n}\right) p_{S_1\emptyset} - (\mathcal{B}_{S_0} + \mathcal{B}_{S_1} + \mathcal{B}^m_{S_0} + \mathcal{B}^m_{S_1})p_{S_0S_1} + 2\mathcal{I}_{S_0}p_{S_0S_0} - \mathcal{I}_{S_0}p_{S_0S_1}\\
&\quad - (2d+\gamma)p_{S_0S_1} + 2\gamma p_{S_1S_1}\\
\frac{dp_{S_0I_0}}{dt} &= \mathcal{H}p_{I_0\emptyset} + 2\mathcal{B}_{S_0}p_{S_0S_0} - \left(\mathcal{B}_{S_0} + \mathcal{B}^m_{S_0} + (1-D)\frac{\beta_0}{n}\right)p_{S_0I_0}\\
&\quad - \left(\mathcal{I}_{S_0} + \mathcal{I}_{I_0} + (1-D)\frac{\omega}{n}\right)p_{S_0I_0} - (2d+\alpha_0)p_{S_0I_0} + \gamma(p_{S_1I_0} + p_{S_0I_1})\\
\frac{dp_{S_0I_1}}{dt} &= \mathcal{H}p_{I_1\emptyset} + \mathcal{B}_{S_1}p_{S_0S_1} - \left(\mathcal{B}_{S_0} + \mathcal{B}^m_{S_0} + (1-D)\frac{\beta_1}{n}\right)p_{S_0I_1} + \mathcal{I}_{I_0}p_{S_0I_0}\\
&\quad - \left(\mathcal{I}_{S_0} + (1-D)\frac{\omega}{n}\right)p_{S_0I_1} - (2d+\alpha_1+\gamma)p_{S_0I_1} + \gamma p_{S_1I_1}\\
\frac{dp_{S_0I_0^m}}{dt} &= \mathcal{H}p_{I_0^m\emptyset} + 2\mathcal{B}^m_{S_0}p_{S_0S_0} - \left(\mathcal{B}_{S_0} + \mathcal{B}^m_{S_0} + (1-D)\frac{\beta_0^m}{n}\right)p_{S_0I_0^m}\\
&\quad - \left(\mathcal{I}_{S_0} + \mathcal{I}_{I_0^m} + (1-D)\frac{\omega}{n}\right)p_{S_0I_0^m} - (2d+\alpha_0^m)p_{S_0I_0^m} + \gamma(p_{S_1I_0^m} + p_{S_0I_1^m})
\end{aligned}$$

$$\frac{dp_{S_0I_1^m}}{dt} = \mathcal{H}p_{I_1^m\emptyset} + \mathcal{B}_{S_1}^m p_{S_0S_1} - \left(\mathcal{B}_{S_0} + \mathcal{B}_{S_0}^m + (1-D)\frac{\beta_1^m}{n}\right) p_{S_0I_1^m} + \mathcal{I}_{I_0^m} p_{S_0I_0^m}$$
$$- \left(\mathcal{I}_{S_0} + (1-D)\frac{\omega}{n}\right) p_{S_0I_1^m} - (2d + \alpha_1^m + \gamma) p_{S_0I_1^m} + \gamma p_{S_1I_1^m}$$

$$\frac{dp_{S_0\emptyset}}{dt} = -\left(\mathcal{H} + (1-D)\frac{b}{n}\right) p_{S_0\emptyset} + 2\mathcal{H}p_{\emptyset\emptyset} - \left(\mathcal{B}_{S_0} + \mathcal{B}_{S_0}^m\right) p_{S_0\emptyset} - \mathcal{I}_{S_0} p_{S_0\emptyset}$$
$$+ d(2p_{S_0S_0} + p_{S_0S_1} + p_{S_0I_0} + p_{S_0I_1} + p_{S_0I_0^m} + p_{S_0I_1^m})$$
$$+ \alpha_0 p_{S_0I_0} + \alpha_1 p_{S_0I_1} + \alpha_0^m p_{S_0I_0^m} + \alpha_1^m p_{S_0I_1^m} - dp_{S_0\emptyset} + \gamma p_{S_1\emptyset}$$

$$\frac{dp_{S_1S_1}}{dt} = -2\left(\mathcal{B}_{S_1} + \mathcal{B}_{S_1}^m\right) p_{S_1S_1} + \mathcal{I}_{S_0} p_{S_0S_1} - 2(d+\gamma) p_{S_1S_1}$$

$$\frac{dp_{S_1I_0}}{dt} = \mathcal{B}_{S_0} p_{S_0S_1} - \left(\mathcal{B}_{S_1} + \mathcal{B}_{S_1}^m + (1-D)\frac{\beta_0}{n}\right) p_{S_1I_0} + \left(\mathcal{I}_{S_0} + (1-D)\frac{\omega}{n}\right) p_{S_0I_0}$$
$$- \mathcal{I}_{I_0} p_{S_1I_0} - (2d + \alpha_0 + \gamma) p_{S_1I_0} + \gamma p_{S_1I_1}$$

$$\frac{dp_{S_1I_1}}{dt} = 2\mathcal{B}_{S_1} p_{S_1S_1} - \left(\mathcal{B}_{S_1} + \mathcal{B}_{S_1}^m + (1-D)\frac{\beta_1}{n}\right) p_{S_1I_1}$$
$$+ \left(\mathcal{I}_{S_0} + (1-D)\frac{\omega}{n}\right) p_{S_0I_1} + \mathcal{I}_{I_0} p_{S_1I_0} - (2d + \alpha_1 + 2\gamma) p_{S_1I_1}$$

$$\frac{dp_{S_1I_0^m}}{dt} = \mathcal{B}_{S_0}^m p_{S_0S_1} - \left(\mathcal{B}_{S_1} + \mathcal{B}_{S_1}^m + (1-D)\frac{\beta_0^m}{n}\right) p_{S_1I_0^m} + \left(\mathcal{I}_{S_0} + (1-D)\frac{\omega}{n}\right) p_{S_0I_0^m}$$
$$- \mathcal{I}_{I_0^m} p_{S_1I_0^m} - (2d + \alpha_0^m + \gamma) p_{S_1I_0^m} + \gamma p_{S_1I_1^m}$$

$$\frac{dp_{S_1I_1^m}}{dt} = 2\mathcal{B}_{S_1}^m p_{S_1S_1} - \left(\mathcal{B}_{S_1} + \mathcal{B}_{S_1}^m + (1-D)\frac{\beta_1^m}{n}\right) p_{S_1I_1^m}$$
$$+ \left(\mathcal{I}_{S_0} + (1-D)\frac{\omega}{n}\right) p_{S_0I_1^m} + \mathcal{I}_{I_0^m} p_{S_1I_0^m} - (2d + \alpha_1^m + 2\gamma) p_{S_1I_1^m}$$

$$\frac{dp_{S_1\emptyset}}{dt} = -\left(\mathcal{H} + (1-D)\frac{b}{n}\right) p_{S_1\emptyset} - (\mathcal{B}_{S_1} + \mathcal{B}_{S_1}^m) p_{S_1\emptyset} + \mathcal{I}_{S_0} p_{S_0\emptyset}$$
$$+ d(p_{S_0S_1} + 2p_{S_1S_1} + p_{S_1I_0} + p_{S_1I_1} + p_{S_1I_0^m} + p_{S_1I_1^m})$$
$$+ \alpha_0 p_{S_1I_0} + \alpha_1 p_{S_1I_1} + \alpha_0^m p_{S_1I_0^m} + \alpha_1^m p_{S_1I_1^m} - (d+\gamma) p_{S_1\emptyset}$$

$$\frac{dp_{I_0I_0}}{dt} = \left(\mathcal{B}_{S_0} + (1-D)\frac{\beta_0}{n}\right) p_{S_0I_0} - 2\left(\mathcal{I}_{I_0} + (1-D)\frac{\omega}{n}\right) p_{I_0I_0} - 2(d+\alpha_0) p_{I_0I_0} + \gamma p_{I_0I_1}$$

$$\frac{dp_{I_0I_1}}{dt} = \left(\mathcal{B}_{S_0} + (1-D)\frac{\beta_1}{n}\right) p_{S_0I_1} + \left(\mathcal{B}_{S_1} + (1-D)\frac{\beta_0}{n}\right) p_{S_1I_0} + 2\left(\mathcal{I}_{I_0} + (1-D)\frac{\omega}{n}\right) p_{I_0I_0}$$
$$- \left(\mathcal{I}_{I_0} + (1-D)\frac{\omega}{n}\right) p_{I_0I_1} - (2d + \alpha_0 + \alpha_1 + \gamma) p_{I_0I_1} + 2\gamma p_{I_1I_1}$$

$$\frac{dp_{I_0I_0^m}}{dt} = \mathcal{B}_{S_0} p_{S_0I_0^m} + \mathcal{B}_{S_0}^m p_{S_0I_0} - \left(\mathcal{I}_{I_0} + \mathcal{I}_{I_0^m} + 2(1-D)\frac{\omega}{n}\right) p_{I_0I_0^m} - (2d + \alpha_0 + \alpha_0^m) p_{I_0I_0^m} + \gamma(p_{I_1I_0^m} + p_{I_0I_1^m})$$

$$\frac{dp_{I_0I_1^m}}{dt} = \mathcal{B}_{S_0} p_{S_0I_1^m} + \mathcal{B}_{S_1}^m p_{S_1I_0} + \left(\mathcal{I}_{I_0^m} + (1-D)\frac{\omega}{n}\right) p_{I_0I_0^m} - \left(\mathcal{I}_{I_0} + (1-D)\frac{\omega}{n}\right) p_{I_0I_1^m}$$
$$- (2d + \alpha_0 + \alpha_1^m + \gamma) p_{I_0I_1^m} + \gamma p_{I_1I_1^m}$$

$$\frac{dp_{I_0\emptyset}}{dt} = -\mathcal{H}p_{I_0\emptyset} + \mathcal{B}_{S_0} p_{S_0\emptyset} - \mathcal{I}_{I_0} p_{I_0\emptyset} + d(p_{S_0I_0} + p_{S_1I_0} + 2p_{I_0I_0} + p_{I_0I_1} + p_{I_0I_0^m} + p_{I_0I_1^m})$$
$$+ 2\alpha_0 p_{I_0I_0} + \alpha_1 p_{I_0I_1} + \alpha_0^m p_{I_0I_0^m} + \alpha_1^m p_{I_0I_1^m} - (d+\alpha_0) p_{I_0\emptyset} + \gamma p_{I_1\emptyset}$$

$$\frac{dp_{I_1I_1}}{dt} = \left(\mathcal{B}_{S_1} + (1-D)\frac{\beta_1}{n}\right) p_{S_1I_1} + \left(\mathcal{I}_{I_0} + (1-D)\frac{\omega}{n}\right) p_{I_0I_1} - 2(d + \alpha_1 + \gamma) p_{I_1I_1}$$

$$\frac{dp_{I_1I_0^m}}{dt} = \mathcal{B}_{S_1} p_{S_1I_0^m} + \mathcal{B}_{S_0}^m p_{S_0I_1} + \left(\mathcal{I}_{I_0} + (1-D)\frac{\omega}{n}\right) p_{I_0I_0^m} - \left(\mathcal{I}_{I_0^m} + (1-D)\frac{\omega}{n}\right) p_{I_1I_0^m}$$
$$- (2d + \alpha_1 + \alpha_0^m + \gamma) p_{I_1I_0^m} + \gamma p_{I_1I_1^m}$$

$$\frac{dp_{I_1I_1^m}}{dt} = \mathcal{B}_{S_1} p_{S_1I_1^m} + \mathcal{B}_{S_1}^m p_{S_1I_1} + \left(\mathcal{I}_{I_0} + (1-D)\frac{\omega}{n}\right) p_{I_0I_1^m} + \left(\mathcal{I}_{I_0^m} + (1-D)\frac{\omega}{n}\right) p_{I_1I_0^m}$$
$$- (2d + \alpha_1 + \alpha_1^m + 2\gamma) p_{I_1I_1^m}$$

$$\frac{dp_{I_1\emptyset}}{dt} = -\mathcal{H}p_{I_1\emptyset} + \mathcal{B}_{S_1} p_{S_1\emptyset} + \mathcal{I}_{I_0} p_{I_0\emptyset} + d(p_{S_0I_1} + p_{S_1I_1} + p_{I_0I_1} + 2p_{I_1I_1} + p_{I_1I_0^m} + p_{I_1I_1^m})$$
$$+ \alpha_0 p_{I_0I_1} + 2\alpha_1 p_{I_1I_1} + \alpha_0^m p_{I_1I_0^m} + \alpha_1^m p_{I_1I_1^m} - (d + \alpha_1 + \gamma) p_{I_1\emptyset}$$

$$\frac{dp_{I_0^mI_0^m}}{dt} = \left(\mathcal{B}_{S_0}^m + (1-D)\frac{\beta_0^m}{n}\right) p_{S_0I_0^m} - 2\left(\mathcal{I}_{I_0^m} + (1-D)\frac{\omega}{n}\right) p_{I_0^mI_0^m} - 2(d + \alpha_0^m) p_{I_0^mI_0^m} + \gamma p_{I_0^mI_1^m}$$

$$
\begin{aligned}
\frac{dp_{I_0^m I_1^m}}{dt} &= \left(\mathcal{B}_{S_0}^m + (1-D)\frac{\beta_1^m}{n}\right) p_{S_0 I_1^m} + \left(\mathcal{B}_{S_1}^m + (1-D)\frac{\beta_0^m}{n}\right) p_{S_1 I_0^m} + \left(\mathcal{I}_{I_0^m} + (1-D)\frac{\omega}{n}\right)(2p_{I_0^m I_0^m} - p_{I_0^m I_1^m}) \\
&\quad - (2d + \alpha_0^m + \alpha_1^m + \gamma) p_{I_0^m I_1^m} + \gamma p_{I_1^m I_1^m} \\
\frac{dp_{I_0^m \emptyset}}{dt} &= -\mathcal{H} p_{I_0^m \emptyset} + \mathcal{B}_{S_0}^m p_{S_0 \emptyset} - \mathcal{I}_{I_0^m} p_{I_0^m \emptyset} + d(p_{S_0 I_0^m} + p_{S_1 I_0^m} + p_{I_0 I_0^m} + p_{I_1 I_0^m} + 2p_{I_0^m I_0^m} + p_{I_1^m I_0^m}) \\
&\quad + \alpha_0 p_{I_0 I_0^m} + \alpha_1 p_{I_1 I_0^m} + 2\alpha_0^m p_{I_0^m I_0^m} + \alpha_1^m p_{I_0^m I_1^m} - (d + \alpha_0^m) p_{I_0^m \emptyset} + \gamma p_{I_1^m \emptyset} \\
\frac{dp_{I_1^m I_1^m}}{dt} &= \left(\mathcal{B}_{S_1}^m + (1-D)\frac{\beta_1^m}{n}\right) p_{S_1 I_1^m} + \left(\mathcal{I}_{I_0^m} + (1-D)\frac{\omega}{n}\right) p_{I_0^m I_1^m} - 2(d + \alpha_1^m + \gamma) p_{I_1^m I_1^m} \\
\frac{dp_{I_1^m \emptyset}}{dt} &= -\mathcal{H} p_{I_1^m \emptyset} + \mathcal{B}_{S_1}^m p_{S_1 \emptyset} + \mathcal{I}_{I_0^m} p_{I_0^m \emptyset} + d(p_{S_0 I_1^m} + p_{S_1 I_1^m} + p_{I_0 I_1^m} + p_{I_1 I_1^m} + p_{I_0^m I_1^m} + 2p_{I_1^m I_1^m}) \\
&\quad + \alpha_0 p_{I_0 I_1^m} + \alpha_1 p_{I_1 I_1^m} + \alpha_0^m p_{I_0^m I_1^m} + 2\alpha_1^m p_{I_1^m I_1^m} - (d + \alpha_1^m + \gamma) p_{I_1^m \emptyset} \\
\frac{dp_{\emptyset\emptyset}}{dt} &= -2\mathcal{H} p_{\emptyset\emptyset} + d(p_{S_0\emptyset} + p_{S_1\emptyset} + p_{I_0\emptyset} + p_{I_1\emptyset} + p_{I_0^m\emptyset} + p_{I_1^m\emptyset}) + \alpha_0 p_{I_0\emptyset} + \alpha_1 p_{I_1\emptyset} + \alpha_0^m p_{I_0^m\emptyset} + \alpha_1^m p_{I_1^m\emptyset}
\end{aligned}
$$

where $\mathcal{H}, \mathcal{B}_X, \mathcal{B}_X^m, \mathcal{I}_X$ represent the rate of host reproduction, transmission rate of resident pathogens, transmission rate of mutant pathogens, and transmission of information, respectively. These rates, in general, depend on the local density around a pair (e.g. $q_{X/YZ}$) and this requires additional information on higher moments of the spatial distribution of sites (e.g. the density of triplets). We used pair approximation to close this system (see Eq. (86)) which yields

$$
\begin{aligned}
\mathcal{H} &= (1-D)\frac{n-1}{n} b(q_{S_0/\emptyset} + q_{S_1/\emptyset}) + Dbp_S \\
\mathcal{B}_X &= (1-D)\frac{n-1}{n}(\beta_0 q_{I_0/X} + \beta_1 q_{I_1/X}) + D(\beta_0 p_{I_0} + \beta_1 p_{I_1}) \\
\mathcal{B}_X^m &= (1-D)\frac{n-1}{n}(\beta_0^m q_{I_0^m/X} + \beta_1^m q_{I_1^m/X}) + D(\beta_0^m p_{I_0} + \beta_1^m p_{I_1}) \\
\mathcal{I}_X &= (1-D)\frac{n-1}{n}\omega(q_{I_0/X} + q_{I_1/X} + q_{I_0^m/X} + q_{I_1^m/X}) + D\omega(p_{I_0} + p_{I_1} + p_{I_0^m} + p_{I_1^m})
\end{aligned}
$$

where n is the number of neighbouring sites. In this paper, we consider the regular square lattice ($n = 4$). We implemented the above 28 equations in a Mathematica notebook in https://doi.org/10.5281/zenodo.20271193.

References

1. SP Otto, T Day, *A biologist's guide to mathematical modeling in ecology and evolution.* (Princeton University Press), (2007).
2. H Caswell, *Matrix population models.* (Sinauer Sunderland, MA) Vol. 1, (2000).
3. PD Taylor, Allele-frequency change in a class-structured population. *The Am. Nat.* **135**, 95–106 (1990).
4. S Lion, M van Baalen, The evolution of juvenile-adult interactions in populations structured in age and space. *Theor. Popul. Biol.* **76**, 132–145 (2009).
5. YH Zurita-Gutiérrez, S Lion, Spatial structure, host heterogeneity and parasite virulence: implications for vaccine-driven evolution. *Ecol. Lett.* **18**, 779–789 (2015).
6. S Lion, Class structure, demography and selection: reproductive-value weighting in nonequilibrium, polymorphic populations. *The Am. Nat.* **191**, 620–637 (2018).
7. S Lion, From the price equation to the selection gradient in class-structured populations: a quasi-equilibrium route. *J. Theor. Biol.* **447C**, 178–189 (2018).
8. S Lion, S Gandon, Evolution of class-structured populations in periodic environments. *Evolution* **76**, 1674–1688 (2022).
9. JA Metz, RM Nisbet, SA Geritz, How should we define 'fitness' for general ecological scenarios? *Trends ecology & evolution* **7**, 198–202 (1992).
10. Y Cai, SA Geritz, Resident-invader dynamics of similar strategies in fluctuating environments. *J. mathematical biology* **81**, 907–959 (2020).
11. SA Geritz, E Kisdi, G Mesze´ NA, JA Metz, Evolutionarily singular strategies and the adaptive growth and branching of the evolutionary tree. *Evol. ecology* **12**, 35–57 (1998).
12. S Alizon, A Hurford, N Mideo, M Van Baalen, Virulence evolution and the trade-off hypothesis: history, current state of affairs and the future. *J. evolutionary biology* **22**, 245–259 (2009).
13. S Lion, JA Metz, Beyond r0 maximisation: on pathogen evolution and environmental dimensions. *Trends ecology & evolution* **33**, 458–473 (2018).
14. J Metz, S Mylius, O Diekmann, When does evolution optimize? *Evol. Ecol. Res.* **10**, 629–654 (2008).
15. N Bacaër, Approximation of the basic reproduction number r 0 for vector-borne diseases with a periodic vector population. *Bull. mathematical biology* **69**, 1067–1091 (2007).
16. J Heesterbeek, M Roberts, Threshold quantities for infectious diseases in periodic environments. *J. biological systems* **3**, 779–787 (1995).
17. VH Khong, P Carmona, S Gandon, Seasonality and the persistence of vector-borne pathogens. *J. Royal Soc. Interface* **20** (2023).

18. M Kamo, A Sasaki, Evolution toward multi-year periodicity in epidemics. *Ecol. Lett.* **8**, 378–385 (2005).
19. S Lion, Moment equations in spatial evolutionary ecology. *J. theoretical biology* **405**, 46–57 (2016).
20. S Lion, Mv Baalen, Self-structuring in spatial evolutionary ecology. *Ecol. letters* **11**, 277–295 (2008).
21. S Lion, M Boots, Are parasites "prudent"in space? *Ecol. Lett.* **13**, 1245–1255 (2010).
22. S Lion, S Gandon, Evolution of spatially structured host–parasite interactions. *J. Evol. Biol.* **28**, 10–28 (2015).
23. M Boots, A Sasaki, 'small worlds' and the evolution of virulence: infection occurs locally and at a distance. *Proc. Royal Soc. London. Ser. B: Biol. Sci.* **266**, 1933–1938 (1999).
24. M Van Baalen, DA Rand, The unit of selection in viscous populations and the evolution of altruism. *J. theoretical biology* **193**, 631–648 (1998).
25. H Matsuda, N Ogita, A Sasaki, K Satō, Statistical mechanics of population: the lattice lotka-volterra model. *Prog. theoretical Phys.* **88**, 1035–1049 (1992).
26. Y Haraguchi, A Sasaki, The evolution of parasite virulence and transmission rate in a spatially structured population. *J. Theor. Biol.* **203**, 85–96 (2000).
27. DT Gillespie, Exact stochastic simulation of coupled chemical reactions. *The journal physical chemistry* **81**, 2340–2361 (1977).
28. JH Gillespie, Natural selection for within-generation variance in offspring number. *Genetics* **76**, 601 (1974).
29. TL Parsons, A Lambert, T Day, S Gandon, Pathogen evolution in finite populations: slow and steady spreads the best. *J. The Royal Soc. Interface* **15**, 20180135 (2018).
